# Revealing the biomolecular response of glioma cells to helium, carbon and oxygen minibeam radiation therapy using synchrotron-based infrared microspectroscopy

**DOI:** 10.1039/d5an01327e

**Published:** 2026-06-22

**Authors:** R. González-Vegas, O. Seksek, S. Chiblak, S. Brons, A. Abdollahi, Y. Prezado, I. Yousef, I. Martínez-Rovira

**Affiliations:** a Physics Department, Universitat Autònoma de Barcelona (UAB) 08193 Cerdanyola del Vallès Barcelona Spain Immaculada.Martinez@uab.cat; b IJCLab, French National Centre for Scientific Research 91450 Orsay France; c Heidelberg Ion-Beam Therapy Center (HIT), Department of Radiation Oncology, Heidelberg University Hospital (UKHD) 69120 Heidelberg Germany; d Clinical Cooperation Unite Translational Radiation Oncology, German Cancer Consortium (DKTK) Core Center, National Center for Tumor Diseases (NCT), Heidelberg University Hospital (UKHD) and German Cancer Research Center (DKFZ) 69120 Heidelberg Germany; e Institut Curie, CNRS UMR3347, Inserm U1021, Signalisation Radiobiologie et Cancer, Université PSL 91400 Orsay France; f CNRS UMR3347, Inserm U1021, Signalisation Radiobiologie et Cancer, Université Paris-Saclay 91400 Orsay France; g New Approaches in Radiotherapy Lab, Center for Research in Molecular Medicine and Chronic Diseases (CIMUS), Instituto de Investigación Sanitaria de Santiago de Compostela (IDIS), University of Santiago de Compostela 15706 Santiago de Compostela A Coruña Spain; h Oportunius Program, Galician Agency of Innovation (GAIN), Xunta de Galicia Santiago de Compostela A Coruña Spain; i Institut Curie Centre de Recherche Rue Henri Becquerel 91410 Orsay France; j MIRAS Beamline, ALBA Synchrotron 08209 Cerdanyola del Vallès Barcelona Spain

## Abstract

Combining helium, carbon or oxygen beams with minibeam radiation therapy (MBRT) may benefit the treatment of radioresistant tumours while better protecting healthy tissues from radiation toxicities. In this study, the biomolecular response of glioma cell lines to HeMBRT, CMBRT and OMBRT was evaluated using synchrotron-based Fourier transform infrared microspectroscopy (SR-FTIRM). F98 (rat glioma) and U-87 MG (human glioma) cell lines were subjected to conventional broad beam RT (BB) or MBRT at the Heidelberg Ion-Beam Therapy Centre (Germany). Biomolecular effects were assessed with SR-FTIRM at the MIRAS beamline of the ALBA Synchrotron (Spain). Principal component analysis (PCA) uncovered the spectral alterations due to the different irradiation modalities. In F98 cells, IR signatures in the 1254–1225 cm^−1^ spectral region, mainly related to DNA and RNA geometries, were altered by both BB and MBRT modalities and the two ion species. Alterations of IR signatures in the 1097–1074 cm^−1^ spectral region, associated with the phosphodiester backbone of nucleic acids, and IR signatures associated with C–O vibrational modes in the 1110–1097 cm^−1^ (mainly due to nucleic acids), 1182–1163 cm^−1^ (mainly due to phospholipids), 1135–1110 cm^−1^ and 1071–1040 cm^−1^ (mainly due to carbohydrates) spectral regions, were generally enhanced by CMBRT; OBB and OMBRT also resulted in dose-dependent modifications of these spectral bands, suggesting nucleic acid modifications or oxidative damage. CMBRT, OBB and OMBRT also induced changes in IR signatures of the Amide I band associated with α-helical and β-sheet protein secondary structures, which might result from protein oxidation or cell death mechanisms. In U-87 MG cells, specific IR signatures in the Phosphate II band (*i.e.* 1173 cm^−1^, 1150 cm^−1^, 1080 cm^−1^, 1065 cm^−1^ and 1025 cm^−1^), primarily associated with C–O signals present in phospholipids, carbohydrates and the phosphodiester backbone of nucleic acids, were greatly affected by helium-, carbon- and oxygen-ion RT, in both conventional and spatially fractionated modes. Biomolecular changes in the C–H vibrational modes of lipids for both cell lines were consistent with free radical attacks. Cell viability results revealed cell line-dependent sensitivities to treatment, with findings consistent with the modifications observed in the SR-FTIRM analysis.

## Abstract 2

The biomolecular impact of helium, carbon and oxygen minibeam radiation therapy on F98 and U-87 MG glioma cell lines is investigated by means of synchrotron radiation-based infrared microspectroscopy.

## Graphical abstract

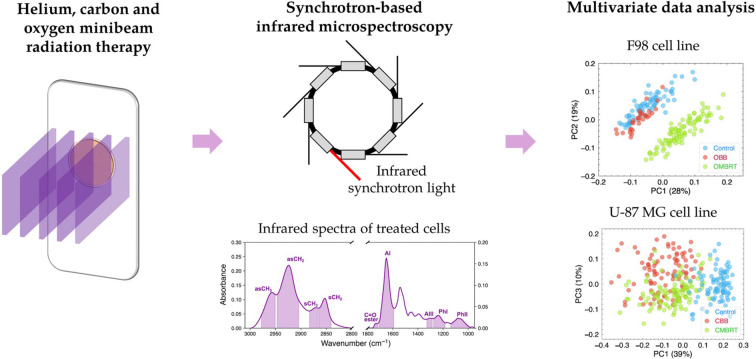

## 1. Introduction

More than 50% of cancer patients receive radiotherapy (RT) at some point during their treatment, making this modality one of the main therapeutic options for cancer care. RT has been the focus of extensive technological innovations aimed at improving treatment outcomes. However, the radiation tolerance of normal tissues remains the main limitation of current treatments. This restriction complicates the treatment of certain radioresistant cancer variants, such as high-grade brain gliomas.

The use of ion beams, such as those of helium, carbon, or oxygen ions, could be a strategy to overcome the limitations of conventional RT approaches. Their enhanced physical and radiobiological properties compared to other particle types used in conventional RT could be of benefit in the treatment of radioresistant tumours. The Heidelberg Ion-Beam Therapy Center (HIT, Heidelberg, Germany) is a dedicated, hospital-based irradiation facility that offers the opportunity to study the application of these promising radiation qualities in RT.^1^ The use of helium beams in RT might be clinically relevant due to their lower multiple Coulomb scattering compared to protons and their reduced nuclear fragmentation tails compared to heavier ions.^2^ Carbon ions are generally considered to be the best particles in terms of physical and radiobiological properties, as they exhibit higher dose conformity and biological effectiveness than X-rays or protons, enhancing tumour control probabilities.^3,4^ Heavier ions, such as oxygen, could also be effective for treating hypoxic tumours due to their reduced oxygen enhancement ratio (OER).^5^

A possible synergy could be achieved through the combination of the aforementioned ion beams with minibeam radiation therapy (MBRT). This novel oncology treatment modality is a spatially fractionated dose-delivery technique: radiation is distributed in a heterogeneous pattern alternating high- and low-dose regions, referred to as peaks and valleys (respectively).^6^ MBRT beams are typically 0.5–1 mm wide, separated by a centre-to-centre (c-t-c) distance of 1–4 mm. The main advantage of MBRT is the significant protection of healthy tissues receiving radiation, as demonstrated in multiple preclinical studies.^7–9^ In addition, MBRT has shown equal or superior tumour control compared to conventional RT.^9–11^ For these reasons, the combination of helium-, carbon-, and oxygen-ion beams with MBRT (HeMBRT, CMBRT, and OMBRT, respectively) might allow for exploiting the superior radiobiological properties of these particles while reducing radiation-induced toxicities to normal tissues;^12–15^ this has already been demonstrated for neon MBRT.^15,16^ These combinations have the potential to overcome the limitations of conventional RT and benefit the treatment of highly radiation-resistant tumours, such as gliomas.

Previous studies have examined the dosimetric feasibility of these techniques. HeMBRT provides improved dose profiles and peak-to-valley dose ratio (PVDR) values compared to proton MBRT at equal c-t-c distances, which might be beneficial for sparing healthy tissues.^14^ Regarding CMBRT and OMBRT, both modalities demonstrate similar dose profiles and very large PVDR values, which are significantly higher than those for X-ray and proton MBRT, suggesting a reduction in normal tissue complication probability as well.^17,18^ In the case of CMBRT, some biological experiments have already been performed.^12,13^ A very recent study observed a similar tumour growth delay for both conventional RT and spatially fractionated carbon RT. Nonetheless, the fact that over 70% of the tumour volume subjected to CMBRT received a valley dose of 1.5 Gy suggests the activation of radiobiological mechanisms that differ from those involved in conventional RT.^13^ Still, the picture of the differential biomolecular response activated by MBRT is not yet fully complete. Some of the mechanisms proposed to explain MBRT efficacy include a significant impact on the immature vasculature,^19^ enhanced repair processes due to the migration of stem cells from valley to peak regions,^20^ activation of the immune system as an anti-tumour response,^21^ cell signalling effects,^22,23^ and the action of reactive oxygen species (ROS).^24^

To unearth deeper insights into the biomolecular effects of HeMBRT, CMBRT and OMBRT modalities, synchrotron-based Fourier transform infrared microspectroscopy (SR-FTIRM) was employed.^25^ SR-FTIRM employs infrared (IR) radiation to interrogate biological samples and reveal their biomolecular structure. Subtle details about RT-induced conformational changes in lipids, proteins, nucleic acids, and carbohydrates can be obtained simultaneously. The high brilliance synchrotron IR radiation allows this type of analysis to be performed at the cellular level. SR-FTIRM has previously been used to analyse the biomolecular basis, both *in vitro* and *in vivo*, that underlies unconventional RT approaches, such as proton therapy,^26^ nanoparticle-enhanced RT,^27–30^ X-ray microbeam RT,^31^ proton and neon MBRT,^32,33^ or FLASH-RT.^34^ One of our previous SR-FTIRM evaluations also provided new insights into the response of an osteosarcoma cell line to CMBRT.^35^

In the present study, SR-FTIRM was employed to further investigate the biomolecular response of glioma cell lines to HeMBRT, CMBRT, and OMBRT. The effects of these novel RT approaches were evaluated for different irradiation doses and compared with the responses to conventional irradiations.

## 2. Experimental section

### 2.1. Sample preparation and irradiations

Rat glioma F98 (CRL-2397™) and human glioma U-87 MG (HTB-14™) cell lines were purchased from the American Type Culture Collection (ATCC®, LGC Standards, Molsheim, France). Both cell lines were grown in high-glucose DMEM (Gibco™, LifeTechnologies, Courtaboeuf, France) supplemented with 10% fetal calf serum, 1% penicillin–streptomycin, 1% glutamine, and 1% sodium pyruvate in an incubation chamber at 37 °C with 5% CO_2_ and 95% humidity. Cells were plated one (F98) or two (U-87 MG) days prior to experimentation to ensure exponential growth; 500 μL of a 4 × 10^5^ cells per mL suspension were seeded in each well of 48-well microplates and incubated overnight to reach 75% confluence before irradiations.

**Fig. 1:**
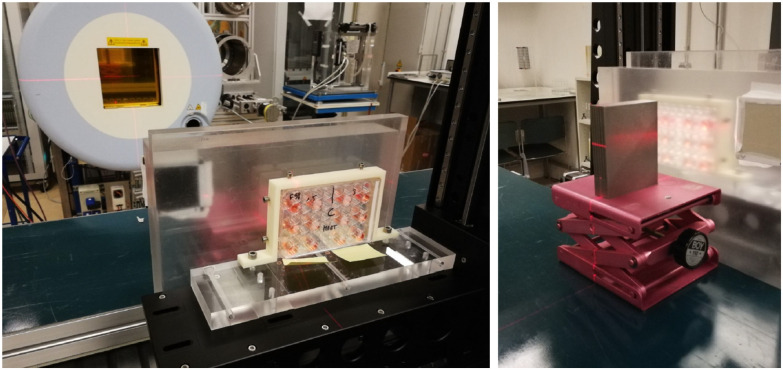
Photograph of the fixed horizontal beam experimental station of HIT, where the irradiation setup for MBRT can be seen. The photograph shows the 48-well microplates containing the samples to irradiate (left) and the multislit tungsten collimator for minibeam generation (right).

RT irradiations were performed at the fixed horizontal beam experimental station of HIT. Both ^12^C and ^16^O ion beams were employed, as well as ^4^He ions for a pilot evaluation on U-87 MG cells. Irradiations were performed in two configurations: conventional broad beam RT (CBB, OBB or HeBB) and MBRT (CMBRT, OMBRT or HeMBRT). In the case of carbon and oxygen irradiations, the following physical mean doses were applied: 1.5 Gy, 5 Gy and 10 Gy. In the case of ^4^He beams, a single physical dose of 10 Gy was evaluated. Minibeams were generated by interposing into the beam a multislit tungsten collimator with five 700 μm-wide line apertures (MBRT peak width), separated by a c-t-c distance of 3500 μm. Cells were placed at the centre of a 2 cm-long spread out Bragg peak (SOBP) region at an 8 cm depth; the total radiation field (15 × 15 mm^2^) was built up using scanned pencil beams. The irradiation setup can be seen in Fig. 1; the setup allows the placement of the well plates vertically while maintaining cells hydrated during irradiations. Radiochromic EBT3 Gafchromic™ films were used to verify irradiation quality.

Sample preparation for SR-FTIRM was carried out in accordance with previous protocols.^27^ Cells were fixed 24 hours after the irradiations to assess the cellular response to treatment. After incubation, the medium containing the samples was removed, and 100 μL of a 0.05% Trypsin-EDTA solution (Life Technologies, Courtaboeuf, France) was added to each well to detach the cells. Next, 500 μL of supplemented DMEM was added, and the cell suspension was centrifuged at 1500 rpm for 5 minutes. The cell pellet was re-suspended in phosphate-buffered saline (PBS) and centrifuged again at 1500 rpm for 5 minutes. The pellet was then re-suspended in 10% neutral formalin buffer (Sigma-Aldrich, Saint-Quentin-Fallavier, France) and incubated for 1 hour at room temperature, then stored at 4 °C. Prior to cytospinning with the Cytospin III (Shandon), samples were centrifuged at 1500 rpm for 5 minutes, and the pellet was rinsed three times in ultrapure Millipore water to remove residual phosphate ions. Cells were then cytospun on 0.5 mm thick infrared CaF_2_ slides at 700 rpm. Lastly, samples were dried at room temperature prior to SR-FTIRM analyses.

### 2.2. SR-FTIRM measurements

SR-FTIRM measurements of Control and RT-treated samples were conducted at the MIRAS beamline of the ALBA Synchrotron. The end-station of the beamline is equipped with a Hyperion 3000 microscope coupled to a Vertex 70 spectrometer (Bruker Optics GmbH, Germany). The 36× Schwarzschild objective and a matching 36× Schwarzschild condenser were used in the transmission operation mode. The microscope is equipped with a liquid nitrogen-cooled mercury cadmium telluride (MCT) detector. A population of single cell spectra was acquired to overcome biological variability and to achieve statistical significance (an average of 70 cells for Control and BB configurations, and an average of 140 cells for MBRT to account for the larger variability within peak and valley regions). However, it is not possible to determine the exact proportion of cells exposed to peak or valley dose regions; therefore, the present analysis reflects the response of the global population, rather than the spatially resolved effects associated with the MBRT dose distributions. Four experimental replicates were considered for the SR-FTIRM measurements. Each cell was manually selected, and IR spectra were recorded with an aperture size of 10 × 10 μm^2^ (IR beam size). Cellular spectra were acquired in the 3000–950 cm^−1^ mid-IR range after 256 co-added scans with a 4 cm^−1^ spectral resolution. To compensate for changes in the ambient conditions (CO_2_ and water vapour levels) in the experimental hutch during data acquisition, background spectra were collected every 10 minutes (corresponding to 10 cell measurements) under the same acquisition parameters as described above.

### 2.3. Data analysis

**Fig. 2:**
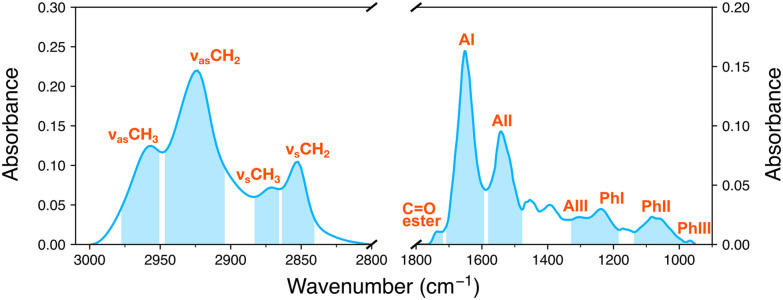
Representative IR absorbance spectrum in the 3000–2800 cm^−1^ (left) and 1800–950 cm^−1^ (right) spectral regions. Blue areas represent the ranges of the most relevant IR bands (indicated by orange labels) in both spectral regions. The spectrum was baseline corrected and vector normalised. Symbols refer to: *ν*: stretching vibration, s: symmetric mode, as: asymmetric mode, A: amide, Ph: phosphate.

Multivariate data analysis was conducted using the open source software Quasar (version 1.11.1).^36^ A representative spectrum showing the main IR bands analysed in this study can be seen in Fig. 2. Assessment of Mie extinction extended multiplicative signal correction (ME-EMSC)^37^ demonstrated no impact on clustering, statistical outcomes, or biological interpretation of the data. Accordingly, Mie scattering effects were considered negligible, and the analyses were conducted on uncorrected data.

Principal component analysis (PCA) allowed us to assess the irradiation-induced spectral modifications and identify the differences between treatment modalities. The principal component (PC) score plots delineate the clustering of samples according to the spectral differences between RT modalities, and the loadings help to identify the main IR signatures that contribute to segregating the data. Two spectral regions were subjected to the PCA:

• Fingerprint (FP, 1467–950 cm^−1^): mainly consisting of complex sugar–phosphate bending and skeletal vibration modes arising from nucleic acids and carbohydrates, which inform about backbone conformations;^38^

• Higher wavenumber (HW, 3000–2800 cm^−1^): originating from asymmetric and symmetric stretching vibrations of the C–H methyl and methylene groups present in the hydrocarbon acyl chains of membrane lipids, providing details on the composition of lipids and the physical state of cell membranes.^39^

The assignments of the main IR peaks in the spectral regions subjected to the PCA can be seen in Table 1. Pre-processing of the IR data prior to the PCA included second-order differentiation using the Savitzky–Golay filter (19-point window for the FP region; 9-point window for the HW region) followed by unit-vector normalisation. Analysis of second-derivative spectral data allowed us to resolve subtle, overlapping IR bands and avoid baseline corrections.^25^ A global PCA was performed to evaluate the relative positioning of all irradiation modalities simultaneously, and the spectral differences between groups were assessed using PCA pairwise comparisons (*i.e.* Control–BB, Control–MBRT, BB–MBRT). The variances explained by each component are specified in each PC scatter plot for the two spectral regions analysed.

**Table 1:** Assignments of the main IR peaks in the HW (3000–2800 cm^−1^) and FP (1467–950 cm^−1^) spectral regions. Wavenumber intervals correspond to the full widths of each peak, obtained by inspection of the second derivatives of absorbance spectra. The biological origin of each peak (functional group and macromolecule) is indicated; if a peak is part of a named spectral band, *e.g.*, the Amide III (AIII), the Phosphate I (PhI), the Phosphate II (PhII), or the Phosphate III (PhIII), the corresponding band is also included in parentheses. Symbols refer to: *ν*: stretching vibration, *δ*: bending vibration, s: symmetric mode, as: asymmetric mode

Wavenumber (cm^−1^)	Assignment	References
2978–2946	*ν*_as_CH_3_ (lipids)	39 and 40
2946–2904	*ν*_as_CH_2_ (lipids)	39 and 40
2884–2865	*ν*_s_CH_3_ (lipids)	39 and 40
2865–2840	*ν*_s_CH_2_ (lipids)	39 and 40
1467–1453	*δ*_s_CH_2_ (lipids, proteins)	40–42
1453–1435	*δ*_as_CH_3_ (lipids, proteins)	40–42
1420–1400	*ν*_s_COO^−^ (proteins, lipids)	39, 41 and 42
1400–1370	*δ*_s_CH_3_ (lipids, proteins)	40–42
1330–1200	*δ*N–H, *ν*C–N, *ν*C=O (proteins; AIII)	41 and 42
1260–1195	*ν*_as_PO_2_^−^ (nucleic acids; PhI)	39 and 42
1182–1163	*ν*_as_C–O (lipids)	40, 42 and 43
1163–1140	*ν*_as_C–O (carbohydrates)	42 and 43
1135–1110	*ν*_as_C–O (carbohydrates; PhII)	39, 42 and 43
1110–1097	*ν*C–O (nucleic acids; PhII)	43 and 44
1097–1074	*ν*_s_PO_2_^−^ (nucleic acids; PhII)	39 and 42
1071–1040	*ν*C–O (carbohydrates, lipids; PhII)	39, 42 and 43
1030–1016	*ν*C–O (carbohydrates; PhII)	38 and 45
1014–990	*ν*/*δ* uracil ring (RNA; PhII)	39 and 46
978–954	*ν*_s_PO_4_^−^ (nucleic acids; PhIII)	38 and 46

Ratios between the areas of specific spectral bands were also assessed and used as markers of biomolecular modifications. Integration of the spectral bands was performed after correcting the baseline of the raw spectra, employing a rubber-band algorithm. The following spectral ratios were considered:

• Asymmetric methylene (2946–2904 cm^−1^) to asymmetric methyl (2978–2946 cm^−1^), denoted as *ν*_as_CH_2_/*ν*_as_CH_3_, and used as a biomarker of the saturation of acyl chains and phospholipid membranes;^40^

• Carbonyl ester (1760–1718 cm^−1^) to asymmetric methyl (2978–2946 cm^−1^), denoted as C=O/*ν*_as_CH_3_, and used as a biomarker of oxidative damage or cell death.^47,48^

The global significance among groups was assessed using the Kruskal–Wallis test. Where differences were statistically significant, a Dunn test (using the Bonferroni correction) was used to perform pairwise comparisons between treatment modalities. Statistical analyses were conducted with the software R (version 4.3.2).^49^

**Fig. 3:**
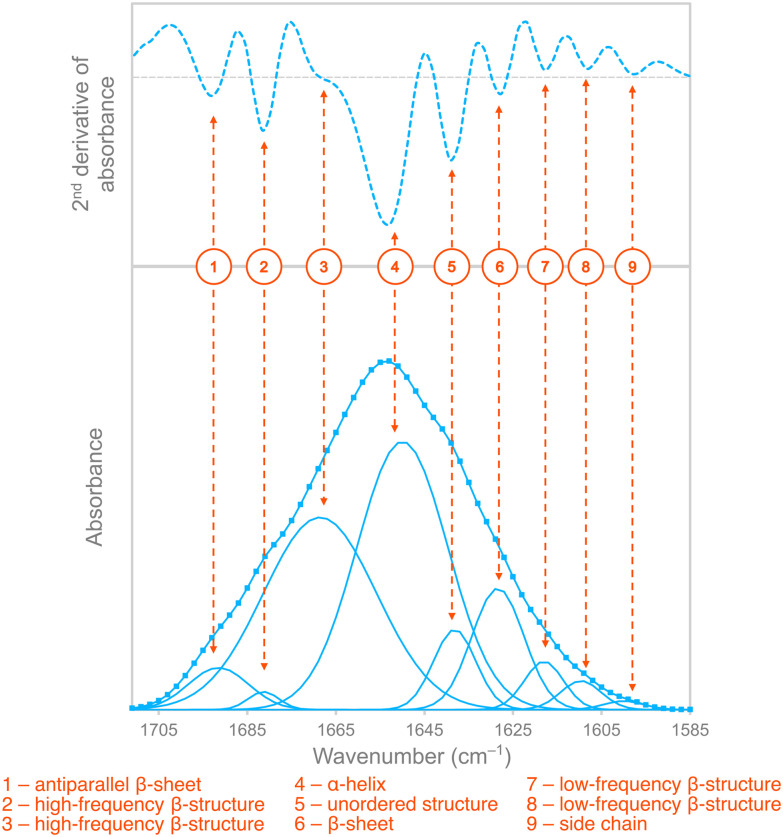
Example of the second derivative of the AI band (top) and results of the peak-fitting analysis (bottom) for one cell. Each sub-band, corresponding to the minima in the 2^nd^ derivative (top) and the continuous blue curves of the fit (bottom), is associated with one of the nine Gaussian functions attributed to the different protein secondary structures indicated in the lower part of the figure. The AI fit, resulting from the sum of each individual sub-band, is indicated by the continuous blue curve with square markers (bottom). The horizontal dashed grey line on the 2^nd^ derivative (top) indicates the origin of the vertical axis.

Additionally, a peak-fitting analysis of the Amide I band (AI, 1711–1585 cm^−1^) was carried out to evaluate changes in the relative secondary structure of proteins.^36,50^ The AI spectral band mainly originates from C=O, C–N and N–H vibrational modes of the proteins and peptides.^41^ Second-order differentiation enabled the assessment of the position of the minima and the number of Gaussian functions to fit the AI band; a linear baseline correction was applied to the AI spectral range before peak fitting. The centres of the Gaussian functions to fit were allowed to vary by 2–8 cm^−1^ and the full width at half maximum (FWHM) by 5–30 cm^−1^.^50^ Nine Gaussian functions associated with different secondary structures of proteins were identified, as shown in Table 2. An example of the peak-fitting analysis of the AI band is depicted in Fig. 3. In the present study, two peaks were identified in the 1684–1657 cm^−1^ spectral range, as well as two additional sub-bands in the 1622–1604 cm^−1^ spectral range. The former peaks could be assigned to high-frequency β-structures (parallel sheets, aggregated strands, turns),^39,41,51–56^ and the latter sub-bands may be ascribed to a different set of low-frequency β-structures (antiparallel and/or parallel sheets, aggregated strands).^39,41,51–57^ It should be noted that there is no general consensus on the assignment of these bands, and their detection may vary depending on the cell line or biological material being analysed and its state. After the peak-fitting analysis, the ratio between the area of the sub-band attributed to β-sheet structures (centred near 1631 cm^−1^) and that of α-helix geometries (centred near 1652 cm^−1^) was evaluated, hereafter denoted as β/α.

**Table 2:** Assignments of AI sub-bands to protein secondary structures. Spectral ranges correspond to the FWHM of the corresponding sub-bands, with the centres of the peaks indicated between brackets

Spectral range [centre] (cm^−1^)	Assignment	References
1700–1684 [1692]	Antiparallel β-sheet	41, 51, 52, 54, 57 and 58
1684–1678 [1681]	High-frequency β-structures	39, 41, 51–56
1687–1657 [1672]		
1665–1639 [1652]	α-helix	39, 41 and 52
1642–1635 [1639]	Unordered structure	39, 41 and 52
1643–1619 [1631]	β-sheet	39, 41 and 52
1622–1614 [1618]	Low-frequency β-structures	39, 41, 51–57
1616–1604 [1610]		
1606–1594 [1600]	Side chain	39, 41, 58 and 59

### 2.4. Cell viability assays

In addition to the SR-FTIRM studies, two complementary procedures were carried out to assess cell viability: a resazurin-based fluorimetric assay to quantify the metabolic activity of living cells, and an apoptosis-dead cell assay based on a fluorogenic substrate for activated caspases (CellEvent™, LifeTechnologies, Courtaboeuf, France). Sample preparation for viability assays was performed under incubation and irradiation conditions identical to those for SR-FTIRM experiments.

Concerning metabolic activity, cells were harvested from each well after irradiations using Trypsin-EDTA treatment, and fresh medium was added to the suspension. Cells were counted using a Countess® II FL Automated Cell Counter (LifeTechnologies, Courtaboeuf, France), and 100 μL of 10^5^ cells per mL were seeded into 96-well microplates. For each treatment condition (irradiation dose and BB or MBRT configurations), 3 wells were prepared. After incubation for 24 hours at 37 °C, 10 μL of a filtered stock solution of resazurin at 0.3 mg mL^−1^ was added to each well, and the microplates were incubated at 37 °C for an additional 2 hours. Next, fluorescence measurements (excitation at 575 nm and emission at 590 nm) were performed using a Fluoroskan Ascent® microplate reader (Thermo Fisher Scientific™, France). The non-irradiated sample was used as a reference to calculate the percentage of metabolically viable cells after treatment.

For the apoptosis-dead cell assay, cells were re-suspended with 100 μL of Trypsin-EDTA and 200 μL of a PBS solution containing the CellEvent™ marker and propidium iodide (final concentration of 5 μM for both labels) and 5% fetal calf serum. Cells were then incubated between 30 minutes and 1 hour at 37 °C. The cells were then centrifuged and re-suspended in 10% neutral formalin buffer. Then, using the Countess® II FL Automated Cell Counter, three images (red for propidium iodide, green for CellEvent™ and *brightfield* for total cell number) were taken for each condition. On each image, cells were counted with a device-related count-to-count variability of 5%. Given the number of live cells counted for the Control sample, dead cells were considered to be the number of red-labeled cells (corresponding to adherent dead cells before trypsinization) added to the difference between the total number of Control cells (*brightfield*) and the total number of cells in the sample (since dead but non-adherent cells could not be visualized, as they were removed before trypsinization). The number of apoptotic cells was measured as the percentage of green-labeled cells over the total number of cells remaining after trypsinization. Values were normalized using the values of the Control group. A typical calculation is as follows: if the total number of cells in the Control (without irradiation) after 24 hours and before trypsinization is *M*_0_ (in cells per mL), and for a certain irradiation modality, the total number of cells drops to *M*_*X*_, then the difference between the total number of cells in the irradiated sample and the Control is (*M*_0_ − *M*_*X*_); this is the number of cells that detached from the support even before trypsinization and due to the irradiation process. The number of cells in apoptosis measured with CellEvent™ is *A*, and the number of cells in necrosis (propidium iodide) is *N*. What is considered the number of “dead” cells for this specific sample is therefore: [(*M*_0_ − *M*_*X*_) + *N*]. The respective percentages of apoptotic and “dead” cells, relative to the Control, are then (*A*/*M*_0_) × 100 and {[(*M*_0_ − *M*_*X*_) + *N*]/*M*_0_} × 100.

## 3. Results and discussion

Results of the data analysis for irradiations with the various ion species are presented in sections 3.1 (F98 cell line) and 3.2 (U-87 MG cell line). The results of the cell viability assays for both cell lines are included in section 3.3.

### 3.1. F98 cell line

#### FP region

**Fig. 4:**
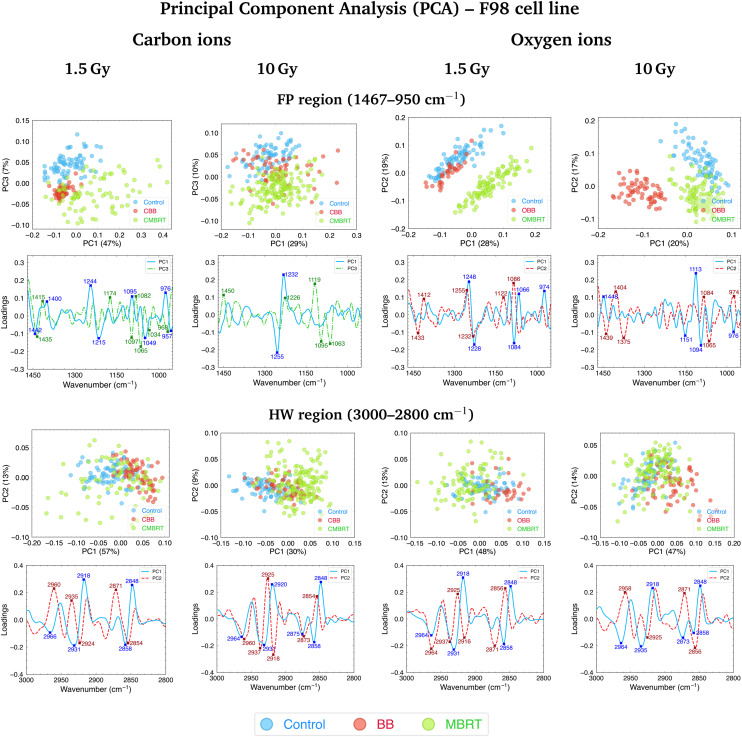
PCA in the FP (1467–950 cm^−1^, top) and HW (3000–2800 cm^−1^, bottom) spectral regions of F98 cells irradiated with carbon (left) or oxygen (right) ions. For each ion species, results for 1.5 Gy (first column) and 10 Gy (second column) irradiations are shown. Each point of the PCA scores represents a cell spectrum, and colours correspond to the irradiation configurations: blue for Control, red for BB and green for MBRT. Variances explained by the PCs are included in parentheses. In the loadings, the contribution of each spectral band to data separation along PC1, PC2 or PC3 is indicated by solid blue lines, dashed red lines or dot-dashed green lines (respectively). The most relevant IR peaks contributing to the cluster delineation along PC1, PC2 or PC3 are indicated with blue, red or green labels and crosses (respectively). For the PCA in the FP region, only the most discriminant projections (*i.e.* PC1–PC2 or PC1–PC3) within the same PCA are included; all the PCA projections for the three doses considered can be found in Fig. S1. Indicated doses refer to the mean dose for both BB and MBRT configurations.

The results of the global PCA in the FP region for the F98 cell line can be seen in Fig. 4 (top; 1.5 Gy and 10 Gy) and Fig. S1 (top). The response of this cell line to irradiation with carbon or oxygen ions presents some differences. In carbon irradiations, CMBRT is generally the most differentiated group from Control cells for the considered doses. For oxygen ions, OMBRT-treated cells are differentiated from Control and OBB-treated cells for the three doses, but OBB was the most dissimilar cluster from non-irradiated cells for 10 Gy irradiations.

The loadings revealed several IR signatures in the 1254–1225 cm^−1^ spectral range contributing to the delineation of data clusters. These signatures are part of the Phosphate I (PhI, 1260–1195 cm^−1^) band, arising mainly from asymmetric PO_2_^−^ stretching vibrations of the sugar–phosphate backbone of nucleic acids,^38^ and the Amide III (AIII, 1330–1200 cm^−1^) band, originating primarily from N–H, C–N and C=O vibrational modes of proteins and polypeptides.^41,42^ Therefore, the peaks indicated by the loadings reflect a complex overlap of spectral contributions, originating from RNA vibrations (1240–1220 cm^−1^),^38,60^ protein secondary structures (*e.g.*, unordered geometries near 1260 cm^−1^, or β-sheets near 1235 cm^−1^),^50^ and from A- and B-form DNA geometries (near 1240 cm^−1^ and 1225 cm^−1^, respectively)^38,60^ and the P=O groups of phospholipids (1240–1220 cm^−1^).^40,43^ PCA pairwise comparisons for carbon irradiations (Fig. S2; top) revealed that these IR signatures contributed to differentiating CBB and CMBRT groups from Control samples for the three doses studied; CMBRT was also associated with changes in these peaks for the lowest dose, resulting in its differentiation from the CBB group. In the case of oxygen treatment (Fig. S3; top), these spectral signatures helped to differentiate OBB and OMBRT clusters from Control cells for the low and intermediate doses; similarly to carbon RT, alterations due to OMBRT for these doses also contributed to the segregation of this group from the OBB cluster. Overall, these spectral modifications, seemingly enhanced by MBRT, may be attributed to RT-induced local conformational changes in the structure of nucleic acids,^44,61^ including DNA condensation and degradation,^62^ RNA upregulation^38,63^ and nucleotide base damage.^44,62^ Other processes, such as protein phosphorylation,^63,64^ oxidative stress^48^ and cell death,^65^ could also explain the observed spectral alterations; some authors also ascribed similar modifications to possible bystander effects.^66^ Furthermore, there is a possibility that irradiation-induced transitions between B-form and A-form DNA geometries may have occurred.^26,38^

Loadings also revealed several IR signatures in the spectral region corresponding to the Phosphate II (PhII, 1140–984 cm^−1^) band. The PhII is comprised of three main contributions: the signatures in the 1135–1110 cm^−1^ spectral range, arising from C–O stretching vibrations of the RNA ribose; the main peak of the PhII band, which is found in the 1097–1074 cm^−1^ spectral region and originates from symmetric PO_2_^−^ stretching vibrations; and the peak in the 1071–1040 cm^−1^ spectral region, which arises from C–O stretching vibrations of the furanose^38^ and symmetric CO–O–C stretching modes of lipids.^39^ As revealed by the PCA pairwise comparisons (Fig. S2 and S3; top), modifications of the previous peaks were associated with the separation of RT-treated cells from the Control groups for both ion species. In addition, the main peak of the PhII band (near 1090 cm^−1^) was primarily associated with modifications due to OMBRT, contributing to its separation from OBB clusters in oxygen irradiations; alterations of this IR signature were also associated with the CBB group for 10 Gy carbon treatment. Furthermore, alterations of the peak in the 1110–1097 cm^−1^ spectral range for RT-treated cells, particularly by MBRT, were observed for both ion species; this peak arises mainly from C–O vibrational modes present in nucleic acids.^43,44^ The overall modifications of the PhII spectral band suggest distinct responses and structural changes in nucleic acids and carbohydrates according to the irradiation modality, ion species, and dose. Such RT-induced alterations, especially by MBRT, may be linked to strand and chromatin fragmentation or cross-links due to irradiations,^44,65^ base stacking and pairing alterations in RNA,^45^ conformational changes and rearrangements in the structure of nucleic acids,^67^ degradation of cell biomembranes,^65^ oxidative damage,^48,68^ and cell death processes.^62^ Other authors also suggested that modifications of the C–O groups present in this spectral region might be ascribed to indirect cell signalling effects.^66,68^ Additionally, the band centred near 1120 cm^−1^ is considered a reliable marker of cellular RNA content.^60^ Changes in this band were mainly associated with modifications due to CMBRT for 5 Gy and 10 Gy carbon irradiations; for treatment with oxygen-ion beams, this peak was primarily associated with alterations due to OBB exposure for the highest dose. These observations suggest enhanced metabolic alterations by the previous RT modalities in a dose-dependent manner.^60^

IR signals in the low-frequency FP region, arising from vibrational modes of the ribose ring (1015–990 cm^−1^) and the nucleic acid backbone (978–954 cm^−1^),^38,46,60^ also contributed to data segregation. Changes in these spectral bands were associated with the separation of RT-treated groups from non-irradiated cells for both ion species (Fig. S2 and S3; top). An exception to this was observed for 10 Gy oxygen irradiations, where alterations of the nucleic acid backbone band were only associated with the segregation of OMBRT-treated cells from Control and OBB clusters. The overall alterations in these low-frequency IR signatures point to enhanced changes in the structure of the ribose–phosphate and deoxyribose backbone due to irradiations, and may result from single- and double-strand breaks, strand cross-links, or deoxyribose damage.^45,61^

Additional spectral signatures in the 1180–1150 cm^−1^ spectral range contributed to the differentiation between some clusters in the PCA pairwise comparisons (Fig. S2 and S3; top). This IR region mainly arises from asymmetric stretching vibrations of the C–O groups present in phospholipids (1182–1163 cm^−1^) and carbohydrates (1163–1140 cm^−1^).^42,43^ Specifically, modifications of the former band were associated with both BB and MBRT modalities in 1.5 Gy and 5 Gy carbon irradiations, and with 1.5 Gy oxygen treatment. In turn, alterations of the latter vibrational mode were associated with the separation of OBB-treated cells from Control and OMBRT groups for 10 Gy oxygen irradiations. Changes in these IR bands suggest an altered lipid and carbohydrate metabolism following irradiations;^62^ along with the previously described modifications of the peak near 1240 cm^−1^, these alterations may be related to an overall cellular response triggered by programmed cell death.^62,65^ Other authors have also proposed that the increased signals near 1180 cm^−1^ and 1240 cm^−1^ might be used as markers of A-form geometries in nucleic acids.^38^

Lastly, the peaks located in the 1462–1375 cm^−1^ spectral range also contributed to cluster delineation. These peaks arise from bending vibrations of the CH_2_ (1467–1453 cm^−1^, symmetric mode) and CH_3_ (1453–1435 cm^−1^, asymmetric mode; 1400–1370 cm^−1^, symmetric mode) groups.^40–42^ Additional contributions within the 1420–1400 cm^−1^ spectral range originate from symmetric stretching modes of carboxylate groups present in amino acid side chains and fatty acids,^41,46^ which may be partially overlapped with the *δ*_s_CH_3_ band. In carbon irradiations, the PCA pairwise comparisons (Fig. S2; top) revealed that modifications of these peaks primarily contributed to the segregation of CMBRT-treated cells from Control and CBB groups. In oxygen-ion RT (Fig. S3; top), both OBB and OMBRT resulted in dose-dependent modifications of these peaks. Alterations of these IR bands may indicate conformational changes in cell membrane lipids, in agreement with the modifications in the 1180–1160 cm^−1^ peak and the C=O ester band (see the results in the HW region sub-section).^42^

Based on the PCA results, the biochemical impact of treatment with carbon or oxygen ions appears to be different. In particular, exposure to oxygen ions generally resulted in a larger cluster delineation than exposure to carbon ions. These differing responses could be due to the distinct radiobiological properties between the radiation species. For example, differences in the linear energy transfer (LET) distributions, relative biological effectiveness (RBE), or oxygen enhancement ratios (OER) might explain the differential impact between carbon and oxygen ions.^5^ Previous studies have observed different levels of RBE values, cell survival, radiation-induced foci and apoptosis following treatment with these radiation species,^4,69–71^ generally enhanced for oxygen beams. Furthermore, differences in ROS production or in the activation of cellular signalling effects between carbon and oxygen ions may also be expected.^5,72^ A prior study observed a differential increase in intracellular ROS levels after carbon and oxygen treatment of a human lung cancer cell line, demonstrating greater ROS accumulation for the latter radiation species;^69^ the production of certain ROS (in particular, of hydrogen peroxide H_2_O_2_) is favoured at higher LET values, which might result in a greater level of indirect cell damage.^24,73,74^ Overall, all these factors might contribute to the differential spectral responses between carbon and oxygen ions in the FP region.

#### HW region

The results of the global PCA in the HW region for carbon and oxygen irradiations are shown in Fig. 4 (bottom; 1.5 Gy and 10 Gy) and Fig. S1 (bottom). For both ion species, BB-treated cells are generally differentiated from the Control cluster. CMBRT-treated cells separate from non-treated samples, whilst OMBRT and Control groups appear closer to each other. This spectral region is dominated by four main bands arising from C–H stretching modes, namely the asymmetric (2978–2946 cm^−1^, *ν*_as_CH_3_) and symmetric (2884–2865 cm^−1^, *ν*_s_CH_3_) methyl bands, and the asymmetric (2946–2904 cm^−1^, *ν*_as_CH_2_) and symmetric (2865–2840 cm^−1^, *ν*_s_CH_2_) methylene bands.^40^ PCA pairwise comparisons for carbon irradiations (Fig. S2; bottom) showed that alterations of the *ν*_as_CH_3_, *ν*_as_CH_2_ and *ν*_s_CH_2_ bands contributed to the segregation of carbon-treated cells from the Control samples; differences in these bands also explained the separation between CBB and CMBRT clusters for 1.5 Gy and 10 Gy irradiations. In addition, differences in the *ν*_s_CH_3_ band also contributed to separating CMBRT-treated cells from non-irradiated samples for the intermediate and high doses. Regarding oxygen treatment, PCA pairwise comparisons (Fig. S3; bottom) revealed that the main modifications in the HW spectral region resulted from OBB exposure, originating from changes in the *ν*_as_CH_3_, *ν*_as_CH_2_ and *ν*_s_CH_2_ bands as well. The segregation of OMBRT-treated cells from Control samples was mainly linked to variations in the methylene bands. The overall changes observed in these spectral bands suggest a conformational disordering of hydrocarbon chains induced by irradiations.^75,76^

**Fig. 5:**
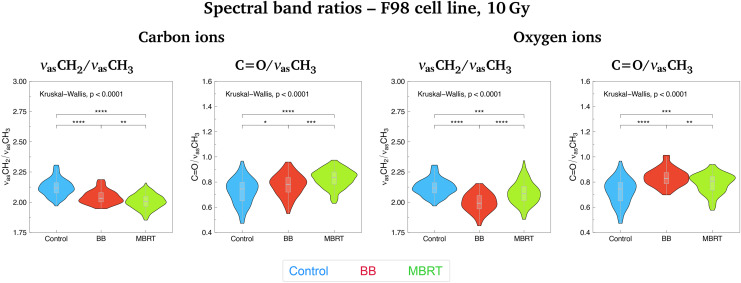
Violin plots showing the probability density distribution of the *ν*_as_CH_2_/*ν*_as_CH_3_ and C=O/*ν*_as_CH_3_ spectral band ratios for F98 cells subjected to 10 Gy carbon (left) or oxygen (right) irradiations. Colours correspond to the irradiation configurations: blue for Control (non-irradiated), red for BB and green for MBRT. *p*-Value significance levels are indicated as: ns (*p* > 0.05), * (*p* ≤ 0.05), ** (*p* ≤ 0.01), *** (*p* ≤ 0.001), **** (*p* ≤ 0.0001).

The *ν*_as_CH_2_/*ν*_as_CH_3_ spectral band ratio was also analysed, and is included in Fig. 5 (first and third columns, 10 Gy) and Fig. S4 (first row, 1.5 Gy and 5 Gy). In carbon irradiations, a reduction of the ratio for irradiated cells with respect to Control samples occurred for all doses, except for the CMBRT group in 1.5 Gy irradiations; still, CMBRT resulted in a greater reduction of the ratio than CBB for the highest dose. Regarding oxygen RT, a decrease in the values of the ratio for OBB-treated cells compared to the values of Control and OMBRT groups was observed for the three doses; exposure to OMBRT with 10 Gy also lowered the ratio relative to the Control sample. The trends of the ratio are akin to the results of the PCA, especially for the highest dose: both CMBRT- and OBB-exposed groups exhibit the greatest spectral alterations. A decrease in the *ν*_as_CH_2_/*ν*_as_CH_3_ ratio was previously ascribed to oxidative stress resulting from ROS attacks. This process could have altered the saturation of the acyl chains and phospholipid structure of cell membranes,^77^ or led to a reduction in their lipid chain lengths.^40^ In addition, previous studies suggested that H_2_O_2_, a radiolytic product originating from water radiolysis, could be one of the main agents accounting for MBRT efficacy.^24,74^ The alterations observed in the methyl and methylene modes could therefore be attributed to the degradation of the cellular lipid structure due to the action of exogenous ROS production.^76^ This behaviour is similar to the effects of CMBRT reported in the LM8 (mouse osteosarcoma) cell line.^35^

Another peak related to the lipid content of cells is the band associated with the ester C=O stretching vibrations present in phospholipid membranes (1760–1718 cm^−1^).^47^ The C=O/*ν*_as_CH_3_ spectral band ratio was therefore analysed and is included in Fig. 5 (second and fourth columns, 10 Gy) and Fig. S4 (second row, 1.5 Gy and 5 Gy). For the two ion species, increased values of the ratio were observed upon BB and/or MBRT treatments, depending on the dose; still, for the highest dose, both irradiation modalities resulted in an increase of the ratio, especially upon CMBRT and OBB. These dose-dependent changes might be a result of cell death processes and enhanced oxidative damage due to the effects of ROS.^47,48^ Other authors ascribed the changes in the C=O ester bonds to a breakage of the hydrogen bonds between the nucleic acid bases, altering their stability.^42^

#### AI peak fitting

**Fig. 6:**
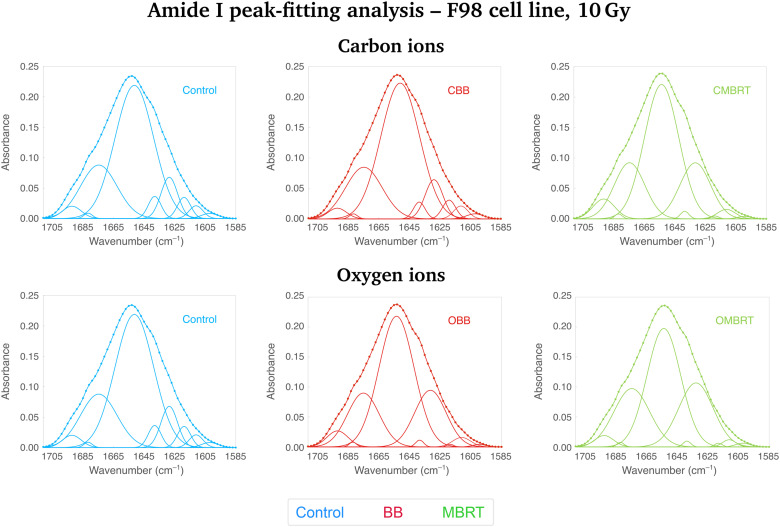
Peak-fitting analysis of the average AI spectral band for the F98 cell line irradiated with 10 Gy carbon (first row) or oxygen (second row) beams. The first to third columns show the fits of the AI band for Control (blue), BB (red) and MBRT (green), respectively. Each sub-band, corresponding to the continuous lines of the fits, is associated with one of the nine Gaussian functions attributed to the different protein secondary structures mentioned in section 2.3. The AI fit, a result of the sum of the nine individual sub-bands, is depicted by continuous curves and square markers.

Changes in the sub-peaks conforming with the AI spectral band, associated with different protein sub-structures, were assessed by performing a peak-fitting analysis.^50,78^ It is worth noting that contributions from RNA, carbohydrate and lipid vibrations are present in the AI spectral range; however, the vibrational modes of the C=O, C–N and N–H groups present in proteins and peptides are expected to be dominant in the AI band.^41^Fig. 6 shows the fits of the average AI spectral band for carbon and oxygen RT. No significant changes in the positions of the sub-bands were detected among the groups. The most striking difference in the intensity of the sub-bands occurred for the one associated with β-sheet secondary structures (1643–1619 cm^−1^, centred near 1631 cm^−1^). An intensity increase of this band was detected in the CMBRT group for 10 Gy irradiations, as well as in OBB- and OMBRT-treated samples for the three doses considered (especially for the latter modality). A decrease in the content of α-helix structures (1665–1639 cm^−1^, centred near 1652 cm^−1^) was also observed for the previous groups, particularly for MBRT. These spectral modifications resulted in an increase in the β/α ratio, which is widely used to analyse the relative protein structure of samples.^79,80^ The evaluation of this ratio was based on the median values obtained from the peak-fitting analysis of all samples in the dataset. For 10 Gy carbon irradiations, the CMBRT group exhibited a relative increase of 93% and 69% compared to Control and CBB-treated cells, respectively. For 10 Gy oxygen irradiations, OBB-treated cells showed a 121% relative increase in the β/α ratio compared with Control cells, while the OMBRT group showed a 236% increase compared with the non-treated sample; for the remaining doses, similar trends were observed. In addition, an increase of the band associated with unordered structures (1642–1635 cm^−1^, centred near 1639 cm^−1^) and a decrease in the low-frequency β-structures (bands in the 1622–1604 cm^−1^ spectral range) were also detected after 10 Gy CMBRT irradiations. Similar changes in these sub-peaks were also seen in oxygen treatments for the three doses considered, especially after OMBRT. The changes observed in these sub-peaks may be associated with modifications in the secondary structures of cellular proteins upon the activation of protein oxidation mechanisms^42^ or cell death processes.^47,81^ Other authors also observed similar modifications and suggested that these may result from deformations of the secondary structure of proteins due to lipid auto-oxidation products,^82^ or from denaturation or redistribution of proteins.^47^ Overall, modifications in the sub-peaks of the AI spectral band appear to be enhanced by 10 Gy CMBRT irradiations, and by both conventional and spatially fractionated oxygen-ion beams (especially in OMBRT-treated samples).

### 3.2. U-87 MG cell line

#### FP region

**Fig. 7:**
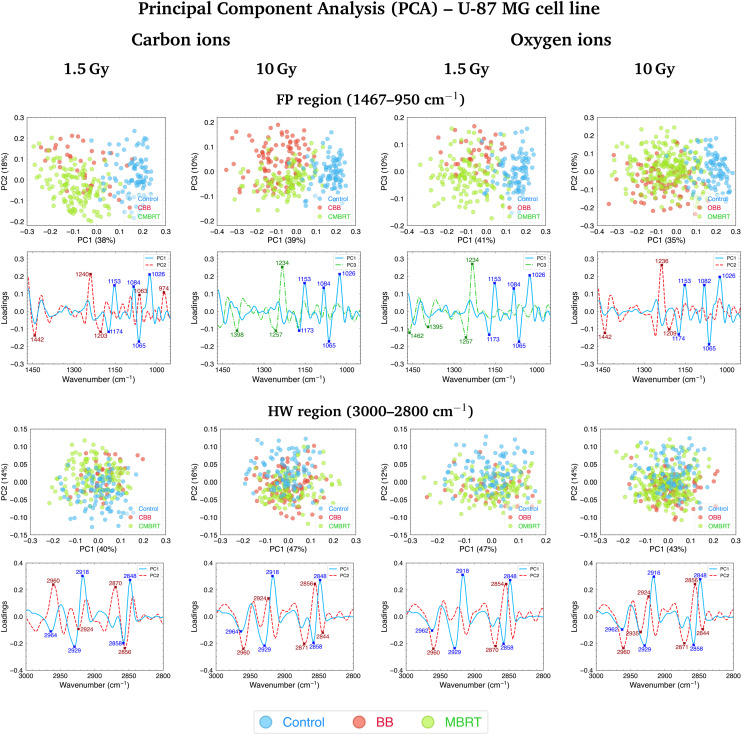
PCA in the FP (1467–950 cm^−1^, top) and HW (3000–2800 cm^−1^, bottom) spectral regions of U-87 MG cells irradiated with carbon (left) or oxygen (right) ions. For each ion species, results for 1.5 Gy (first column) and 10 Gy (second column) irradiations are shown. Each point of the PCA scores represents a cell spectrum, and colours correspond to the irradiation configurations: blue for Control, red for BB and green for MBRT. Variances explained by the PCs are included in parentheses. In the loadings, the contribution of each spectral band to data separation along PC1, PC2 or PC3 is indicated by solid blue lines, dashed red lines or dot-dashed green lines (respectively). The most relevant IR peaks contributing to the cluster delineation along PC1, PC2 or PC3 are indicated with blue, red or green labels and crosses (respectively). For the PCA in the FP region, only the most discriminant projections (*i.e.* PC1–PC2 or PC1–PC3) within the same PCA are included; all the PCA projections for the three doses considered can be found in Fig. S5. Indicated doses refer to the mean dose for both BB and MBRT configurations.

**Fig. 8:**
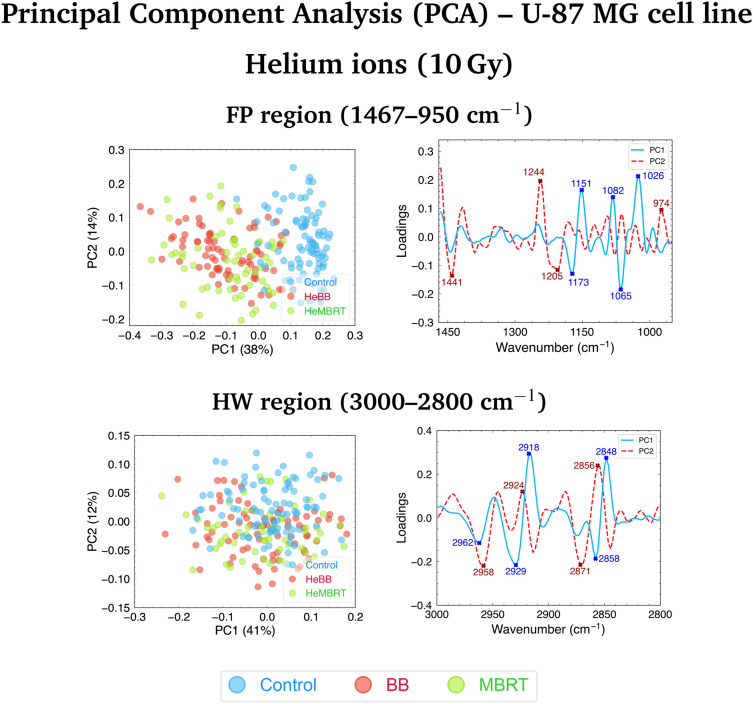
PCA in the FP (1467–950 cm^−1^, first row) and HW (3000–2800 cm^−1^, second row) spectral regions of U-87 MG cells irradiated with helium ions. Each point of the PCA scores represents a cell spectrum, and colours correspond to the irradiation configurations: blue for Control, red for BB and green for MBRT. Variances explained by the PCs are included in parentheses. In the loadings, the contribution of each spectral band to data separation along PC1 or PC2 is indicated by solid blue lines or dashed red lines (respectively). The most relevant IR peaks contributing to the cluster delineation along PC1 or PC2 are indicated with blue or red labels and crosses (respectively). For the PCA in the FP region, only the most discriminant projection (*i.e.* PC1–PC2) within the same PCA is included; all the PCA projections can be found in Fig. S5. A single mean dose of 10 Gy was studied for both BB and MBRT modalities.

The results of the global PCA in the FP region of U-87 MG cells submitted to carbon and oxygen irradiations can be seen in Fig. 7 (top; 1.5 Gy and 10 Gy) and Fig. S5 (top); the results of the pilot evaluation using helium ions can be seen in Fig. 8 (first row) and Fig. S5 (top). The main separation of the data occurs between treated and non-treated cells along PC1 for the three ion species (although differences can be also seen between BB and MBRT clusters). PCA pairwise comparisons (Fig. S6–S8; top) revealed several peaks that consistently contributed to the differentiation between Control and RT-treated samples. On the one hand, the peaks near 1174 cm^−1^ and 1153 cm^−1^ arise from asymmetric stretching vibrations of the C–O groups present in phospholipids and carbohydrates, respectively.^42,43^ Alterations of these IR signatures may reflect an altered carbohydrate metabolism following irradiations,^62^ or conformational alterations of the phospholipid structure;^42^ some authors also suggested that changes in these bands may be related to the activation of cell death mechanisms.^44,62,83^ On the other hand, the peak near 1080 cm^−1^ mainly arises from symmetric stretching vibrations of the phosphodiester backbone of nucleic acids,^38^ and may also include contributions from the phosphate groups of phospholipids.^39,43^ Also, the IR signals near 1065 cm^−1^ and 1026 cm^−1^, contributing the most to data segregation, arise from C–O vibrational modes in carbohydrates and lipids.^38,39,43^ The changes in these IR signatures suggest a different set of conformational alterations induced by irradiations, which may originate from strand breaks or chromatin fragmentation,^61,84^ nucleic acid degradation, condensation or base alterations,^45,62^ or oxidative damage.^46,48^

Additionally, the IR peaks described in the previous paragraph have been previously associated with the carbohydrate profile of glycolipids and glycoproteins.^64,85,86^ Glycogen accumulation was previously observed in tumour cell lines, including the U-87 MG model, under stress conditions such as hypoxia^43^ or following irradiations.^87^ Radiotherapy-induced stress may activate a modulation of the metabolism in tumour cells, particularly glioblastoma, reprogramming the glycolytic activity to promote DNA damage repair responses or reducing the intracellular ROS levels.^88–90^ Therefore, the contribution of the aforementioned carbohydrate IR signals to the separation between RT-treated and Control samples could also reflect an increase in glycogen levels associated with a metabolic adaptation in response to RT. In carbon irradiations (Fig. S6; top), these IR peaks also contributed to the differentiation between CBB and CMBRT groups, suggesting a higher impact on these signatures by the latter modality.

While no additional spectral differences between clusters for helium irradiations were detected (Fig. S8; top), other IR signatures also contributed to differentiating between data clusters for carbon and oxygen treatment. PCA pairwise comparisons for carbon irradiations (Fig. S6; top) showed that several IR signatures in the 1250–1228 cm^−1^ spectral range, corresponding to the PhI and AIII bands, were associated with the separation of carbon-treated cells from Control samples for the three doses considered, especially for the CBB group; CMBRT also induced modifications in these signatures for the highest dose. In addition, the peak near 1210 cm^−1^ contributes to the differentiation between CBB and CMBRT groups; this peak has been previously ascribed to ribose vibrational modes in glioblastoma cells,^43^ but some authors also considered this peak a marker for Z-form DNA.^38^ Regarding oxygen irradiations (Fig. S7; top), the previous spectral signatures contributed to the segregation of both OBB and OMBRT groups from non-treated cells for all the doses considered; the separation between OBB and OMBRT groups was also explained by differences in these IR peaks, primarily associated with alterations due to OMBRT. Previous authors suggested that these spectral modifications may result from high-order DNA degradation,^62^ nucleic acid strand breaks or base cleavage reactions,^83^ oxidative damage,^42^ or even changes in cellular enzymes involved in DNA repair mechanisms.^91^ Additionally, IR signatures assigned to asymmetric CH_3_ bending vibrations (1453–1435 cm^−1^) were mainly associated with the effects of CMBRT (Fig. S6; top); similar modifications also contributed to the separation of both OBB and OMBRT clusters from Control cells for 1.5 Gy and 5 Gy (Fig. S7; top). These spectral changes might be indicative of enhanced conformational alterations of lipid cell membranes.^42^

Compared to the F98 cell line, the response of the U-87 MG cell line to ion-beam RT appears to be different; in particular, only some of the IR signatures contributing to the differences between Control and irradiated cells in the U-87 MG cell line were observed in the PCA of the F98 cell line (primarily the signatures near 1080 cm^−1^ and 1065 cm^−1^). This may be due to the distinct nature of the F98 and U-87 MG cell lines. For instance, previous studies found that these *in vitro* models differed in terms of cell aggregation^92,93^ or migration.^94^ Furthermore, differences in clonogenic survival were observed in a study assessing the sensitivity of these cell lines to both BB and MBRT irradiations.^95^ All these factors could have resulted in the distinct responses to treatment modalities at the biomolecular level.

#### HW region

The PCA in the HW region of U-87 MG cells for carbon and oxygen irradiations can be seen in Fig. 7 (bottom; 1.5 Gy and 10 Gy) and Fig. S5 (bottom). The results for helium ions are shown in Fig. 8 (second row). A separation is observed between irradiated groups and Control samples for the three ion species and all the doses studied. PCA pairwise comparisons (Fig. S6–S8; bottom) revealed alterations in the four methyl and methylene bands present in this spectral region, contributing to the separation of RT-treated samples from Control cells for the three ion species. No remarkable differences between BB and MBRT modalities were detected for any of the ion species.

**Fig. 9:**
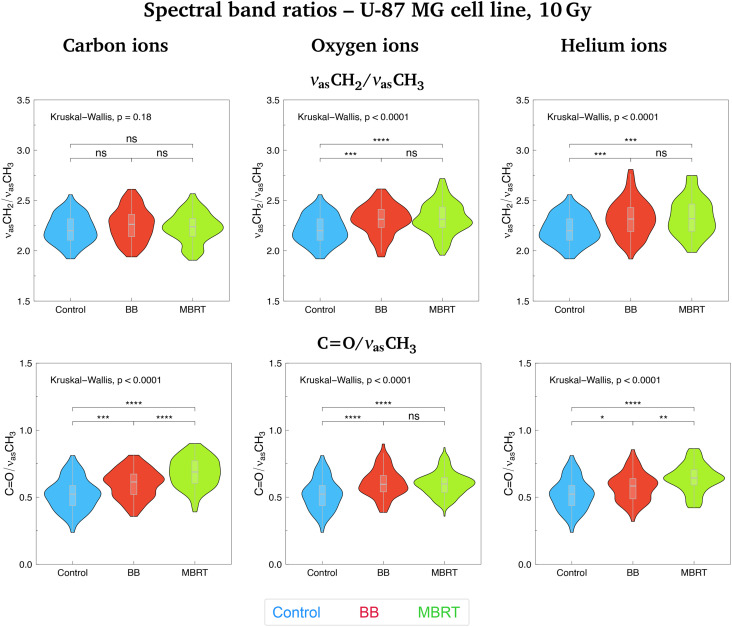
Violin plots showing the probability density distribution of the *ν*_as_CH_2_/*ν*_as_CH_3_ (first row) and C=O/*ν*_as_CH_3_ (second row) spectral band ratios for U-87 MG cells subjected to 10 Gy carbon (first column), oxygen (second column) or helium (third column) irradiations. Colours correspond to the irradiation configurations: blue for Control (non-irradiated), red for BB and green for MBRT. *p*-Value significance levels are indicated as: ns (*p* > 0.05), * (*p* ≤ 0.05), ** (*p* ≤ 0.01), *** (*p* ≤ 0.001), **** (*p* ≤ 0.0001).

The *ν*_as_CH_2_/*ν*_as_CH_3_ ratio, depicted in Fig. 9 (first row, 10 Gy) and Fig. S4 (third row, 1.5 Gy and 5 Gy), was evaluated to elicit further biochemical modifications in this spectral region. In the case of carbon treatments, increases in the values of the ratio were detected for CMBRT-treated cells compared to non-irradiated samples in 1.5 Gy and 5 Gy irradiations. For oxygen and helium RT, significant increases in the ratio for irradiated groups compared to non-irradiated samples were observed. Regarding the C=O/*ν*_as_CH_3_ spectral ratio, shown in Fig. 9 (second row, 10 Gy) and Fig. S4 (fourth row, 1.5 Gy and 5 Gy), a significant increase in the values for irradiated groups compared to Control cells was detected for all ion-beam types. In the case of oxygen beams, no statistical differences were observed between OBB and OMBRT modalities (except for the intermediate dose). In contrast, CMBRT and HeMBRT resulted in a greater increase of the ratio than CBB and HeBB, respectively.

The overall changes in the methyl, methylene, and carbonyl ester vibrational modes in the U-87 MG cell line are similar for the three ion species, generally enhanced for spatially fractionated irradiations. The observed modifications might be related to enhanced oxidative damage due to the action of ROS after treatment^47^ or the activation of cell death processes.^48^ These results are also in agreement with our previous data using proton and neon-ion beams.^32,33^

#### AI peak fitting

**Fig. 10:**
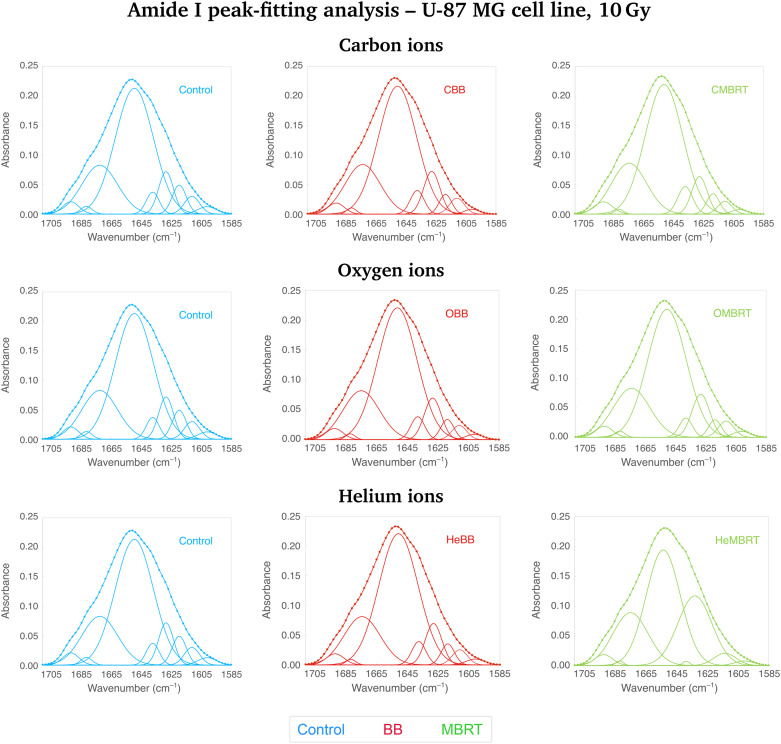
Peak-fitting analysis of the average AI spectral band for the U-87 MG cell line irradiated with 10 Gy carbon (first row), oxygen (second row) or helium (third row) beams. The first to third columns show the fits of the AI band for Control (blue), BB (red) and MBRT (green), respectively. Each sub-band, corresponding to the continuous lines of the fits, is associated with one of the nine Gaussian functions attributed to the different protein secondary structures mentioned in section 2.3. The AI fit, a result of the sum of the nine individual sub-bands, is depicted by continuous curves and square markers.

The results of the AI peak-fitting analysis for the U-87 MG cell line can be seen in Fig. 10. On the one hand, HeMBRT resulted in an intensity increase of the sub-peak assigned to β-sheet structures and a decrease in the sub-bands assigned to α-helical geometries, unordered structures, and low-frequency β-components compared to the Control and HeBB modalities. These modifications by HeMBRT resulted in a relative increase of 238% in the β/α ratio compared to Control and HeBB-treated cells. Similar changes were observed after oxygen treatment: for 10 Gy irradiations, the β/α ratio of OMBRT-treated cells increased by a 62% and 40% relative to Control and OBB groups, respectively (calculations based on the median values of the ratio obtained from the peak-fitting analysis of all samples in the dataset). Similar trends were observed for 1.5 Gy and 5 Gy oxygen irradiations. Previous studies that observed similar spectral modifications ascribed them to an aggregation of proteins with β-sheet structures after oxidation or cell death processes.^42,47,81^ On the other hand, few changes were detected in the different sub-peaks of the AI band after carbon irradiations.

### 3.3. Cell viability assays

**Fig. 11:**
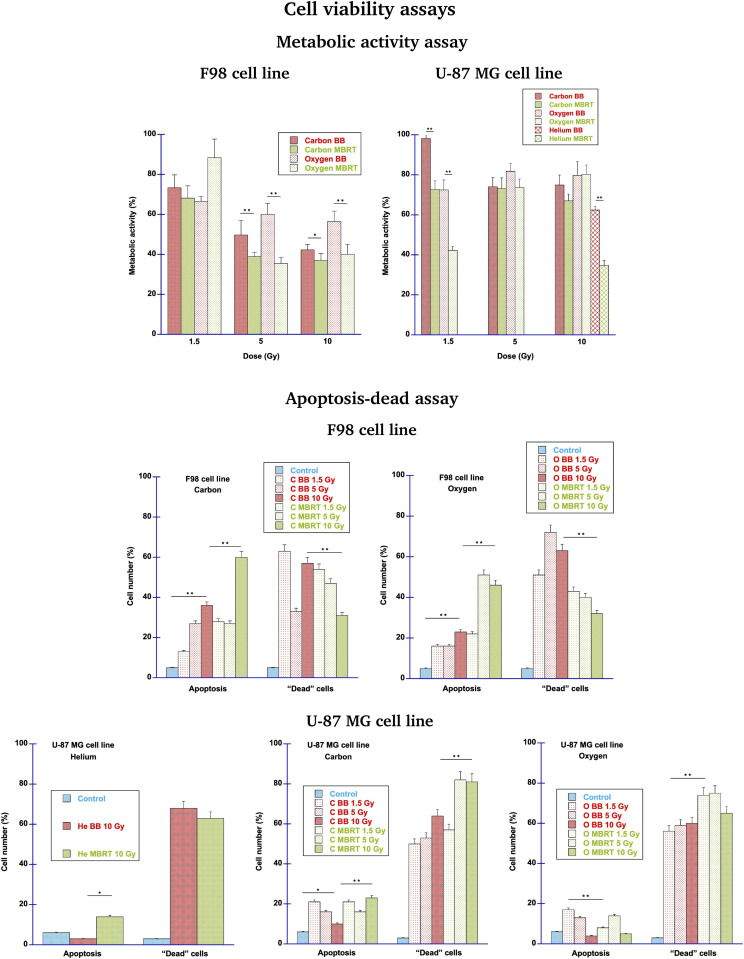
Results of the metabolic activity (first row) and apoptosis-dead (second and third rows) cell viability assays. In the metabolic activity assay, red or green bars represent the percentage of cell viability for BB or MBRT configurations, respectively. For the apoptosis-dead assay, blue bars correspond to the Control group, red bars to BB-treated cells, and green bars to samples subjected to MBRT; percentages of apoptosis or necrosis and detached cells are indicated separately. Data are reported as: mean ± standard deviation. Statistical comparisons were carried out using a one-way ANOVA test, and significant differences were indicated with * (*p* < 0.05) and ** (*p* < 0.01).

Cell viability was assessed 24 hours after irradiations to ensure consistency with the SR-FTIRM measurements and to allow irradiated cells to progress through the first post-treatment cell cycle. The results of the metabolic activity assay and apoptosis-dead cell assay are presented in Fig. 11.

Metabolic activity in F98 cells decreased significantly with dose for both carbon and oxygen ions (Fig. 11, first row). For carbon irradiations, viability dropped to 49.7 ± 7.3% (CBB) and 38.9 ± 2.1% (CMBRT) at 5 Gy, and to 42.3 ± 2.7% (CBB) and 36.0 ± 3.5% (CMBRT) at 10 Gy. A similar trend was observed for oxygen ions, with OMBRT consistently inducing lower residual metabolic activity than OBB (*e.g.*, 56.5 ± 5.2% for OBB and 40.1 ± 5.0% for OMBRT in 10 Gy irradiations). These findings parallel the biomolecular alterations observed by SR-FTIRM in section 3.1, notably the modifications in nucleic acid- and protein-related spectral bands, the relative increase in oxidative damage markers (*e.g.*, the C=O/*ν*_as_CH_3_ ratio), and the alterations in lipid vibrational modes. U-87 MG cells maintained relatively high metabolic activity (around 70–80%) under most conditions (Fig. 11, first row). Nonetheless, viability in U-87 MG cells was reduced under specific conditions, particularly for HeMBRT, which produced a marked decrease (34.7 ± 2.6%) compared to HeBB (62.4 ± 1.7%). This effect is consistent with the pronounced protein secondary-structure alterations observed by SR-FTIRM for helium ions (Fig. 10), including the probable increased β-sheet content and elevated β/α ratio.

The apoptosis-dead cell assay (Fig. 11, second and third rows) confirmed these trends. F98 cells showed a dose-dependent increase in apoptosis, in agreement with the SR-FTIRM signatures of nucleic acid fragmentation and oxidative stress. In U-87 MG cells, apoptosis levels remained moderate but consistently higher than in non-irradiated samples, reflecting the biochemical perturbations detected by SR-FTIRM despite the limited impact on metabolic activity. For all irradiation conditions, a substantial fraction of “dead” cells corresponded to detached cells, which, given the 24 hours of delay, are reasonably considered non-viable detached cells.

The combined analysis of viability assays and SR-FTIRM yields two main conclusions. First, protein and nucleic acid damage is associated with reduced metabolic activity and increased cell death, as shown by the correlation between viability loss (Fig. 11) and the spectral modifications in the FP region and the AI band. Second, oxidative damage, evidenced by changes in lipid and carbonyl vibrational modes, contributes to early viability loss, particularly under MBRT. The enhanced spectroscopic signatures of oxidative stress in MBRT-treated samples are consistent with their generally greater biological effectiveness across ion species.

## 4. Conclusions

This study employed SR-FTIRM as an analytical tool to investigate the effects of HeMBRT, CMBRT and OMBRT on F98 and U-87 MG glioma cell lines, comparing them with the biomolecular response to conventional broad beam irradiations. The use of multivariate analysis methods revealed the main spectral differences between Control, BB- and MBRT-treated samples at 24 hours post-irradiation. This fixation time-point was selected to capture biologically relevant responses, including DNA repair, oxidative stress, and metabolic changes, while avoiding transient effects within the first few hours. It is then expected that most of the changes reported in the manuscript represent the cumulative action of radiation-induced damage and cellular responses. These results provide further insights into the differential biological effects of MBRT combined with carbon, oxygen and helium ions, compared to conventional ion-beam RT.

PCA revealed that the IR signatures of F98 cells exposed to carbon RT were clearly different from those of non-irradiated cells; generally, CMBRT was the most dissimilar group when compared to the Control cells. Structural alterations of specific signatures in the 1467–950 cm^−1^ spectral region contributed to data segregation, mainly associated with the phosphodiester groups of nucleic acids, phospholipids and proteins (1254–1225 cm^−1^ and 1097–1074 cm^−1^), and the C–O vibrational modes present mainly in nucleic acids (1110–1097 cm^−1^), phospholipids (1182–1163 cm^−1^) and carbohydrates (1135–1110 cm^−1^ and 1071–1040 cm^−1^). Alterations of these IR signatures were suggestive of nucleic acid condensation and degradation, cross-links, protein phosphorylation, and other metabolic and conformational alterations in cellular macromolecules, generally enhanced by CMBRT. The effects of this treatment modality on the C–H vibrational modes of lipids, as well as those of high-dose CMBRT on IR signatures associated with protein secondary structures, might result from protein oxidation and cell death processes. Regarding oxygen irradiations, both OBB and OMBRT modalities induced important spectral alterations for the different doses analysed, similar to those reported for carbon irradiations. For the highest dose, the effects of OBB, mainly associated with alterations of spectral signatures related to carbohydrates (peak near 1150 cm^−1^) and nucleic acids (peaks near 1113 cm^−1^ and 1090 cm^−1^) resulted in the segregation of this treatment configuration from the Control and OMBRT groups. Regarding protein-related sub-bands, oxygen-ion treatment affected the contents of α-helical geometries, β-components, and unordered structures, especially in groups subjected to spatially fractionated oxygen beams. RT-induced alterations of the C–H vibrational modes of lipids, especially due to OBB, were suggestive of increased oxidative damage.

As for the U-87 MG cell line, the IR changes due to carbon and oxygen irradiations allowed their differentiation from Control cells, regardless of the irradiation modality (BB or MBRT). The main modifications due to the various treatments were suggestive of an altered carbohydrate and phospholipid metabolism, nucleic acid degradation and oxidative damage. IR signatures possibly related to glycogen accumulation (*i.e.* peaks near 1173 cm^−1^, 1150 cm^−1^, 1080 cm^−1^, 1065 cm^−1^ and 1025 cm^−1^), as previously reported in similar models, were also detected; only some of these signatures contributed to data segregation in the analysis of the F98 cell line. Additionally, differences in the 1250–1228 cm^−1^ IR signatures also contributed to the separation of RT-treated samples from Control cells, especially for the CBB and OMBRT groups. These modifications could originate from irradiation-induced alterations of the structure of nucleic acids, carbohydrates and/or glycoproteins, suggesting a different degree of structural modifications or degradation of the nucleic acids, strand fragmentation, changes in cellular enzymes or oxidative damage. Alterations of methyl, methylene and carbonyl ester bands after BB or MBRT were also consistent with an increased degree of oxidative stress. The results of the PCA from the pilot evaluation with helium-ion beams were in line with the response of U-87 MG samples to carbon and oxygen irradiations. Regarding protein-related spectral bands, few changes were observed after carbon irradiations. Conversely, spectral modifications of β-components, α-helical geometries and unordered structures, mainly due to HeMBRT and OMBRT, suggested the activation of cell death mechanisms and/or protein oxidation processes.

In addition to the molecular insights provided by SR-FTIRM, biological assays performed 24 h post-irradiations reinforce and contextualise the spectroscopic findings. The resazurin-based metabolic activity assay and the apoptosis-dead cell quantification confirmed that the biochemical alterations detected by SR-FTIRM data analysis translate into early functional consequences for cell survival. In F98 cells, spatially fractionated RT induced a greater reduction in viability than conventional irradiations. In the case of U-87 MG cells, a significant vulnerability to specific treatments was also observed. In particular, HeMBRT resulted in a considerable reduction in viability compared to HeBB-exposed cells. Overall, these trends parallel the IR hallmarks of structural perturbations observed in nucleic acids, phospholipids, and proteins. The apoptosis assay also demonstrated an increase in programmed cell death in F98 samples, in a dose-dependent manner. In the case of the U-87 MG cell line, a moderate but consistent apoptotic response was also observed. Overall, these biological endpoints validate that the molecular damage detected by SR-FTIRM may reflect a biologically relevant cytotoxicity.

Finally, SR-FTIRM has proven to be an excellent tool for elucidating the underlying biological mechanisms of novel RT modalities. Further *in vivo* studies are required to provide mechanistic insights at the tissue level.

## Conflicts of interest

The authors have no conflicts of interest to disclose.

## Supplementary Material

AN-151-D5AN01327E-s001

AN-151-D5AN01327E-s002

AN-151-D5AN01327E-s003

AN-151-D5AN01327E-s004

AN-151-D5AN01327E-s005

AN-151-D5AN01327E-s006

AN-151-D5AN01327E-s007

AN-151-D5AN01327E-s008

AN-151-D5AN01327E-s009

AN-151-D5AN01327E-s010

AN-151-D5AN01327E-s011

## Data Availability

Research data will be stored and made available in the Catalan Open Research Area (CORA) research data repository (https://dataverse.csuc.cat). Supplementary information (SI): supplementary projections of the global PCA, PCA pairwise comparisons, spectral band ratios for 1.5 Gy and 5 Gy. See DOI: https://doi.org/10.1039/d5an01327e.
