# Supplementary material for: Revealing the biomolecular response of glioma cells to helium, carbon and oxygen minibeam radiation therapy using synchrotron-based infrared microspectroscopy
Source: Analyst. 2026 Jun 22;151(15):4424–42. doi: 10.1039/d5an01327e (PMC13285976; doi:10.1039/d5an01327e)
Supplement: AN-151-D5AN01327E-s002 [file AN-151-D5AN01327E-s002.pdf]

Supplementary Information (SI) for Analyst.
This journal is © The Royal Society of Chemistry 2026

Supplementary Information

Revealing the biomolecular response of glioma cells to helium, carbon and oxygen minibeam radiation therapy using synchrotron-based infrared microspectroscopy

Roberto González-Vegas[1], Olivier Seksek[2], Sara Chiblak[3,4], Stephan Brons[3], Amir Abdollahi[3,4], Yolanda Prezado[5,6,7,8,9], Ibraheem Yousef[10], and Immaculada Martínez-Rovira[1,*]

[1] *Physics Department, Universitat Autònoma de Barcelona (UAB), 08193 Bellaterra, Cerdanyola del Vallès, Barcelona, Spain*

[2] *IJCLab, CNRS/IN2P3, Université Paris-Saclay, 91450 Orsay, France*

[3] *Heidelberg Ion-Beam Therapy Center (HIT), Department of Radiation Oncology, Heidelberg University Hospital (UKHD), 69120 Heidelberg, Germany*

[4] *Clinical Cooperation Unite Translational Radiation Oncology, German Cancer Consortium (DKTK) Core Center, National Center for Tumor Diseases (NCT), Heidelberg University Hospital (UKHD) and German Cancer Research Center (DKFZ), 69120 Heidelberg, Germany*

[5] *Institut Curie, CNRS UMR3347, Inserm U1021, Signalisation Radiobiologie et Cancer, Université PSL, 91400 Orsay, France*

[6] *CNRS UMR3347, Inserm U1021, Signalisation Radiobiologie et Cancer, Université Paris-Saclay, 91400 Orsay, France*

[7] *New Approaches in Radiotherapy Lab, Center for Research in Molecular Medicine and Chronic Diseases (CIMUS), Instituto de Investigación Sanitaria de Santiago de Compostela (IDIS), University of Santiago de Compostela, Santiago de Compostela, A Coruña, Spain*

[8] *Oportunius Program, Galician Agency of Innovation (GAIN), Xunta de Galicia, 15706 Santiago de Compostela, A Coruña, Spain*

[9] *Institut Curie Centre de Recherche, Rue Henri Becquerel, 91410 Orsay, France*

[10] *MIRAS Beamline, ALBA-CELLS Synchrotron, 08209 Cerdanyola del Vallès, Barcelona, Spain*

[*] Corresponding author. E-mail address: `Immaculada.Martinez@uab.cat`

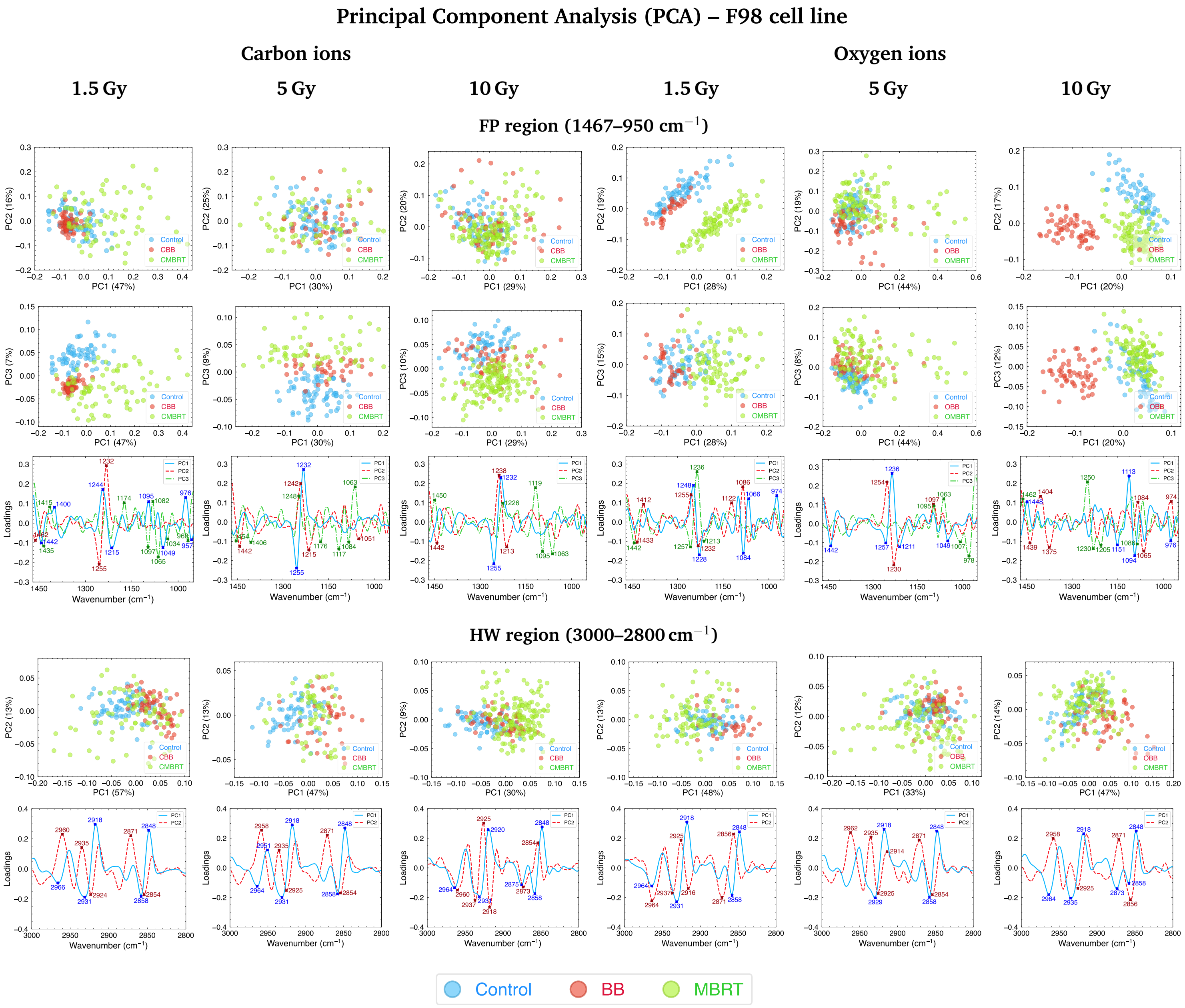

Figure S1. PCA in the FP (1467–950 cm^{-1}, top) and HW (3000–2800 cm^{-1}, bottom) spectral regions of F98 cells irradiated with carbon (left) or oxygen (right) ions. For each ion species, results for 1.5 Gy (first column), 5 Gy (second column) and 10 Gy (third column) are shown. Each point of the PCA scores represents a cell spectrum, and colours correspond to the irradiation configurations: blue for Control, red for BB and green for MBRT. PC1-PC2 and PC1-PC3 score plots are representations of two different factorial planes corresponding to the same PCA. Variances explained by the PCs are included in parentheses. In the loadings, the contribution of each spectral band to data separation along PC1, PC2 or PC3 is indicated by solid blue lines, dashed red lines or dot-dashed green lines (respectively). The most relevant IR peaks contributing to the cluster delineation along PC1, PC2 or PC3 are indicated with blue, red or green labels and crosses (respectively). Indicated doses refer to the mean dose for both BB and MBRT configurations.

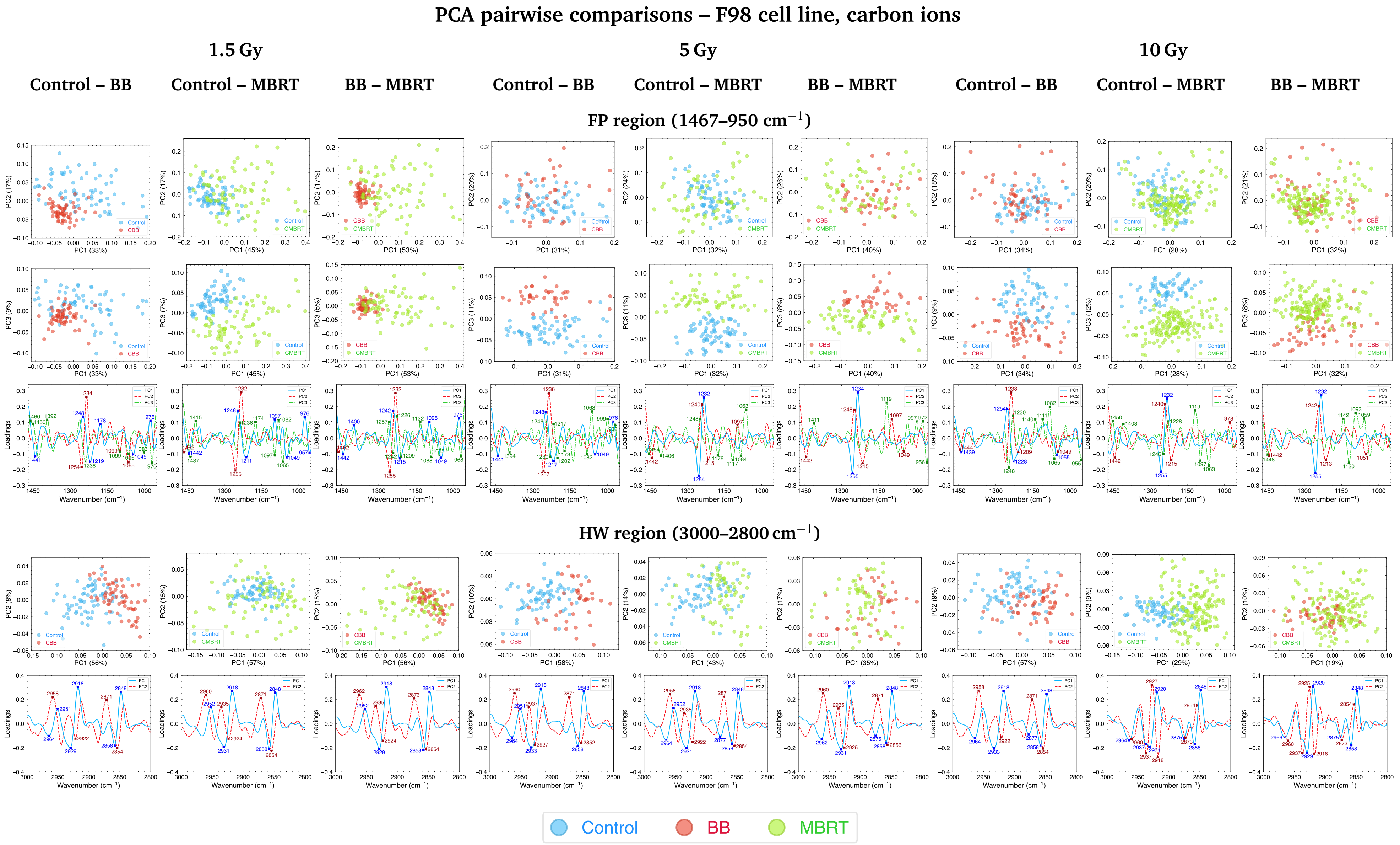

Figure S2. Pairwise PCA in the FP (1467–950 cm^{-1}, top) and HW (3000–2800 cm^{-1}, bottom) spectral regions of F98 cells subjected to 1.5 Gy (left), 5 Gy (centre) and 10 Gy (right) carbon irradiations. Each point of the PCA scores represents a cell spectrum, and colours correspond to the irradiation configurations: blue for Control, red for BB and green for MBRT. For each dose, Control–BB (first column), Control–MBRT (second column) and BB–MBRT (third column) pairwise analyses are included. Variances explained by the PCs are included in parentheses. In the loadings, the contribution of each spectral band to data separation along PC1, PC2 or PC3 is indicated by solid blue, red lines or green lines (respectively). The most relevant IR peaks contributing to the cluster delineation along PC1, PC2 or PC3 are indicated with blue, red or green labels and crosses (respectively). Indicated doses refer to the mean dose for both BB and MBRT configurations.

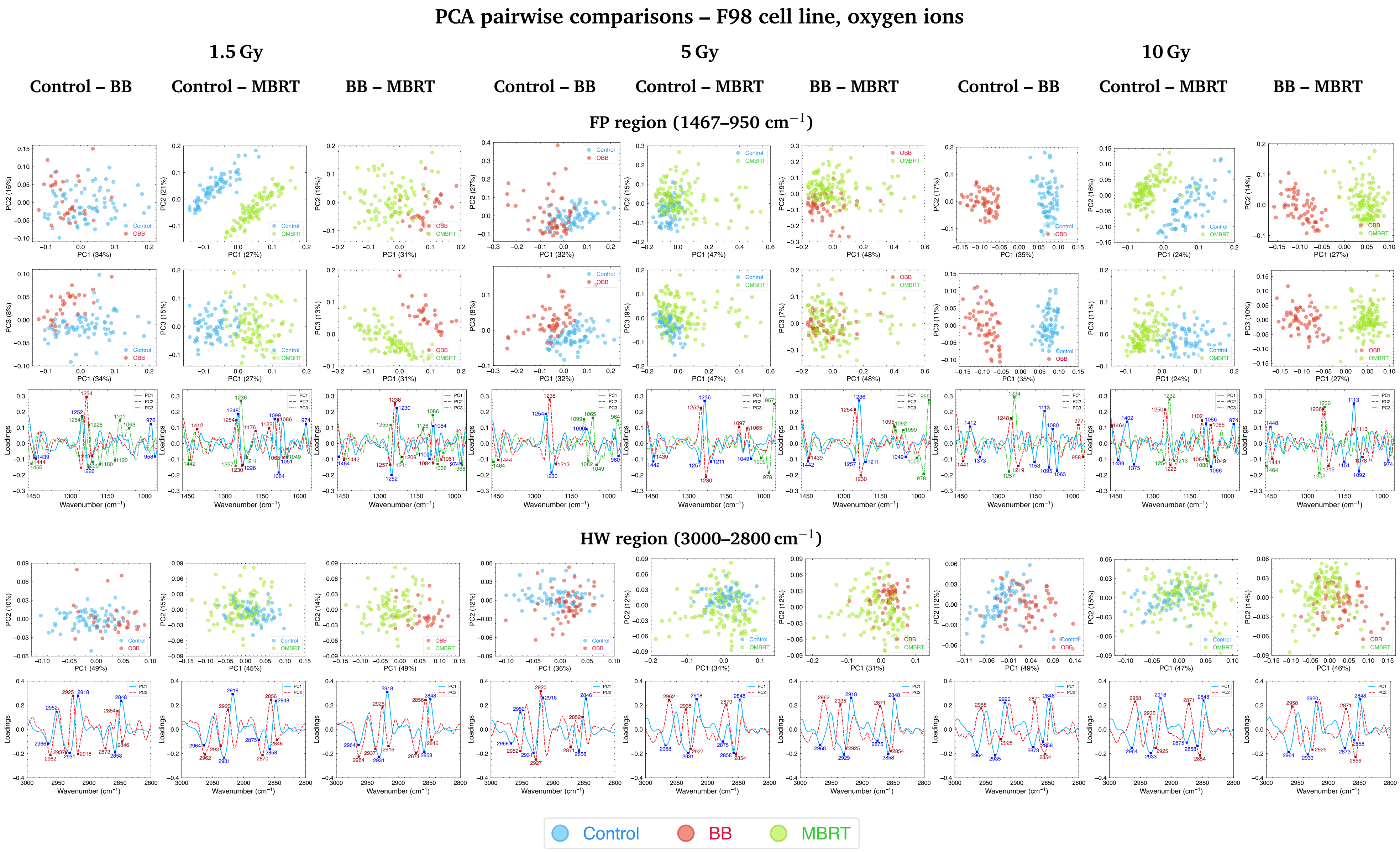

Figure S3. Pairwise PCA in the FP (1467–950 cm^{-1}, top) and HW (3000–2800 cm^{-1}, bottom) spectral regions of F98 cells subjected to 1.5 Gy (left), 5 Gy (centre) and 10 Gy (right) oxygen irradiations. Each point of the PCA scores represents a cell spectrum, and colours correspond to the irradiation configurations: blue for Control, red for BB and green for MBRT. For each dose, Control–BB (first column), Control–MBRT (second column) and BB–MBRT (third column) pairwise analyses are included. Variances explained by the PCs are included in parentheses. In the loadings, the contribution of each spectral band to data separation along PC1, PC2 or PC3 is indicated by solid blue, red lines or green lines (respectively). The most relevant IR peaks contributing to the cluster delineation along PC1, PC2 or PC3 are indicated with blue, red or green labels and crosses (respectively). Indicated doses refer to the mean dose for both BB and MBRT configurations.

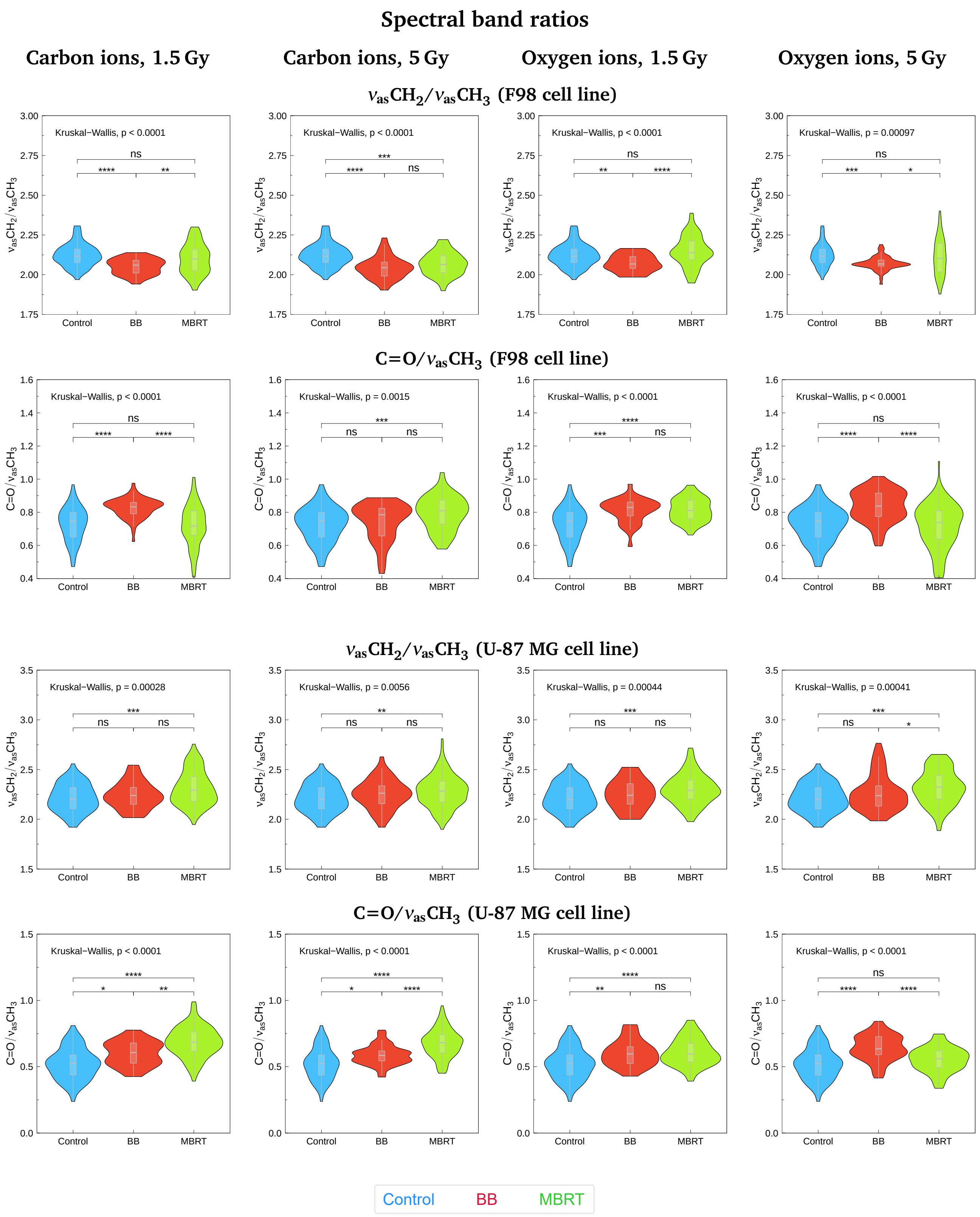

Figure S4. Violin plots showing the probability density distribution of the $\nu_{as}CH_2/\nu_{as}CH_3$ (first and third rows) and $C{=}O/\nu_{as}CH_3$ (second and fourth rows) spectral band ratios for F98 (top) and U-87 MG (bottom) cell lines. Results for 1.5 Gy (first column) or 5 Gy (second column) carbon irradiations and 1.5 Gy (third column) and 5 Gy (fourth column) oxygen irradiations are included. Colours correspond to the irradiation configurations: blue for Control (non-irradiated), red for BB and green for MBRT. p-value significance levels are indicated as: ns ($p > 0.05$), * ($p \leq 0.05$), ** ($p \leq 0.01$), *** ($p \leq 0.001$), **** ($p \leq 0.0001$).

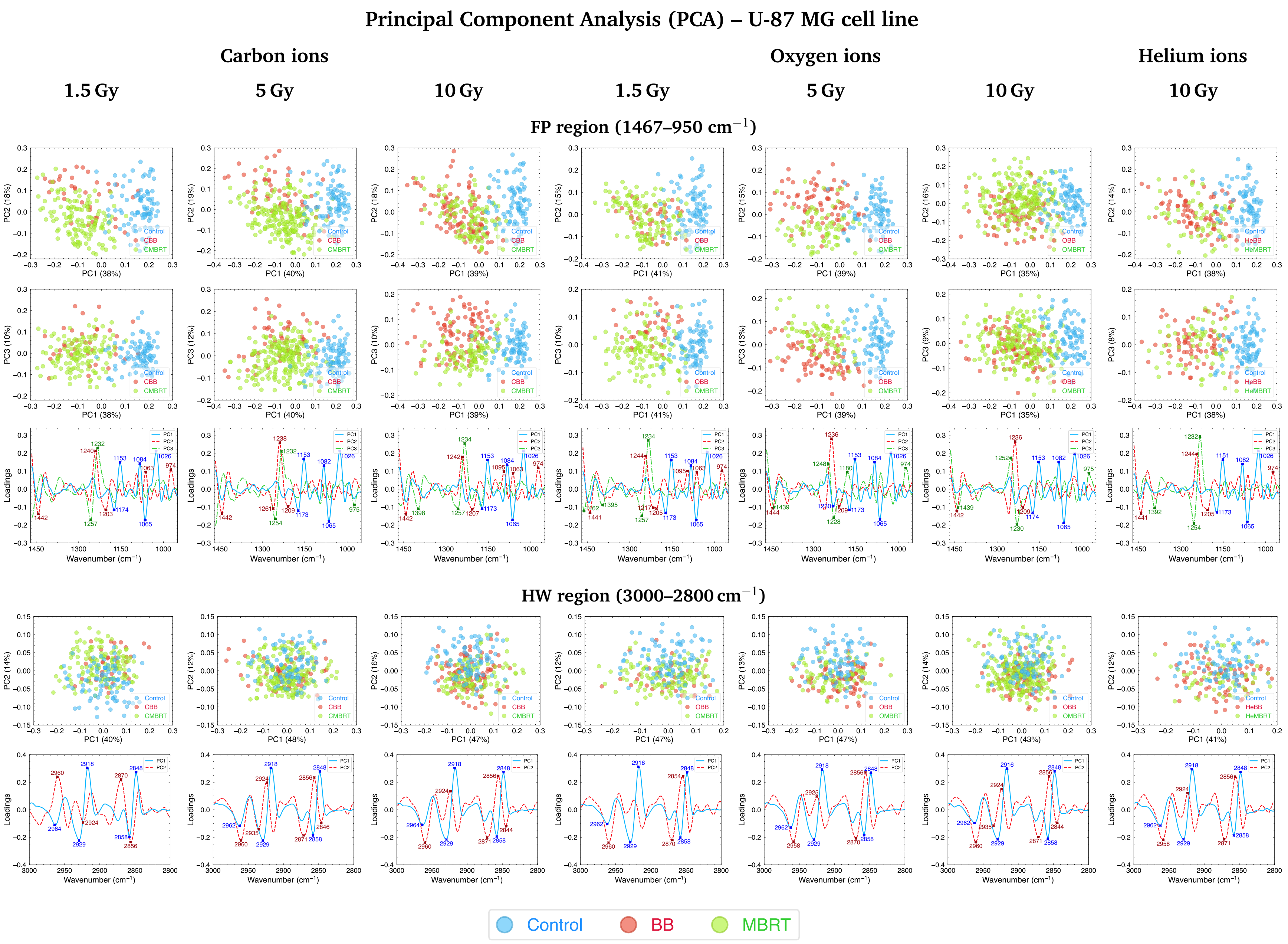

Figure S5. PCA in the FP (1467–950 cm^{-1}, top) and HW (3000–2800 cm^{-1}, bottom) spectral regions of U-87 MG cells irradiated with carbon (left), oxygen (centre) or helium (right) ions. For carbon and oxygen ions, results for 1.5 Gy (first column), 5 Gy (second column) and 10 Gy (third column) are shown. Each point of the PCA scores represents a cell spectrum, and colours correspond to the irradiation configurations: blue for Control, red for BB and green for MBRT. PC1-PC2 and PC1-PC3 score plots are representations of two different factorial planes corresponding to the same PCA. Variances explained by the PCs are included in parentheses. In the loadings, the contribution of each spectral band to data separation along PC1, PC2 or PC3 is indicated by solid blue lines, dashed red lines or dot-dashed green lines (respectively). The most relevant IR peaks contributing to the cluster delineation along PC1, PC2 or PC3 are indicated with blue, red or green labels and crosses (respectively). Indicated doses refer to the mean dose for both BB and MBRT configurations.

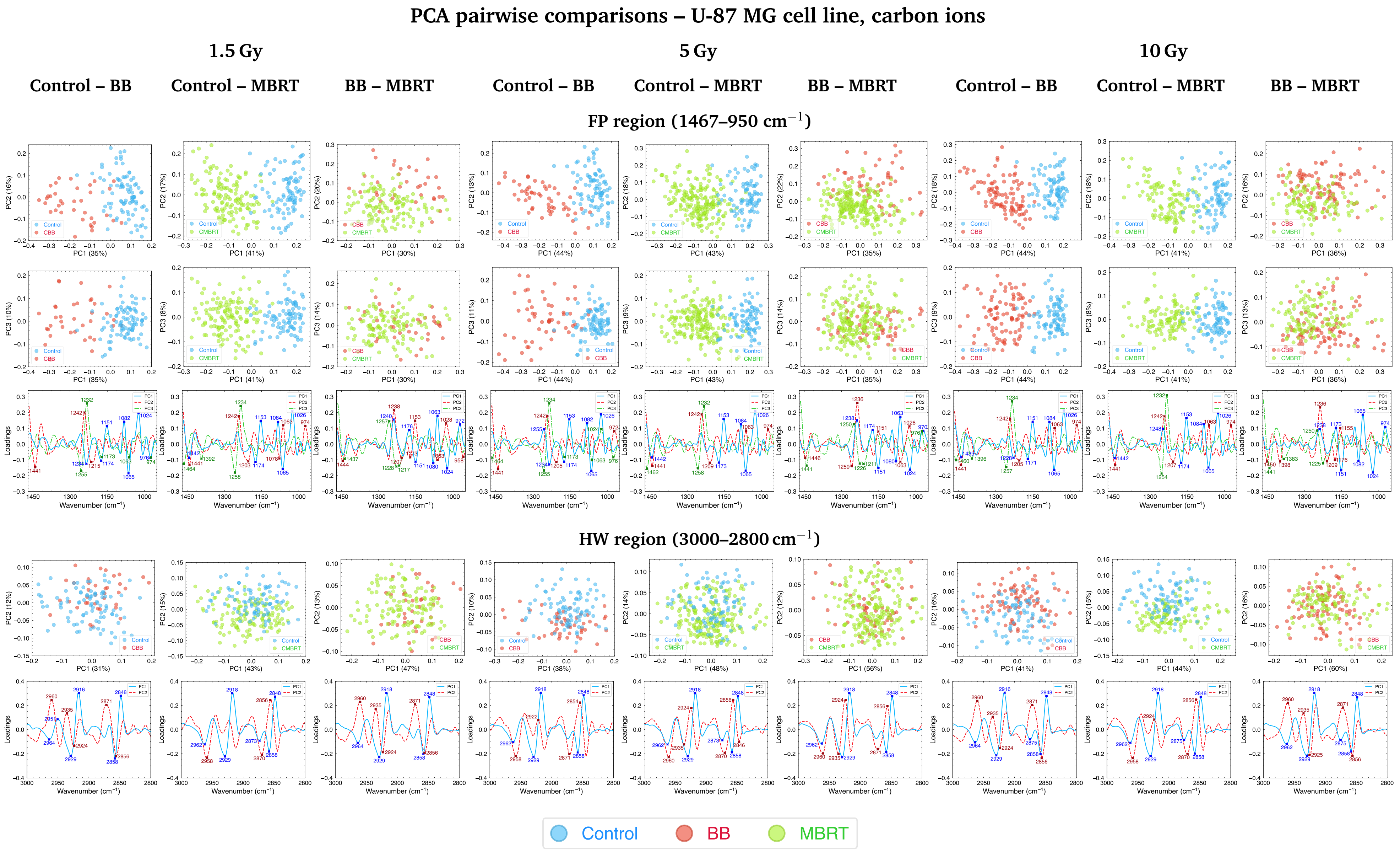

Figure S6. Pairwise PCA in the FP (1467–950 cm^{-1}, top) and HW (3000–2800 cm^{-1}, bottom) spectral regions of U-87 MG cells subjected to 1.5 Gy (left), 5 Gy (centre) and 10 Gy (right) carbon irradiations. Each point of the PCA scores represents a cell spectrum, and colours correspond to the irradiation configurations: blue for Control, red for BB and green for MBRT. For each dose, Control–BB (first column), Control–MBRT (second column) and BB–MBRT (third column) pairwise analyses are included. Variances explained by the PCs are included in parentheses. In the loadings, the contribution of each spectral band to data separation along PC1, PC2 or PC3 is indicated by solid blue, red lines or green lines (respectively). The most relevant IR peaks contributing to the cluster delineation along PC1, PC2 or PC3 are indicated with blue, red or green labels and crosses (respectively). Indicated doses refer to the mean dose for both BB and MBRT configurations.

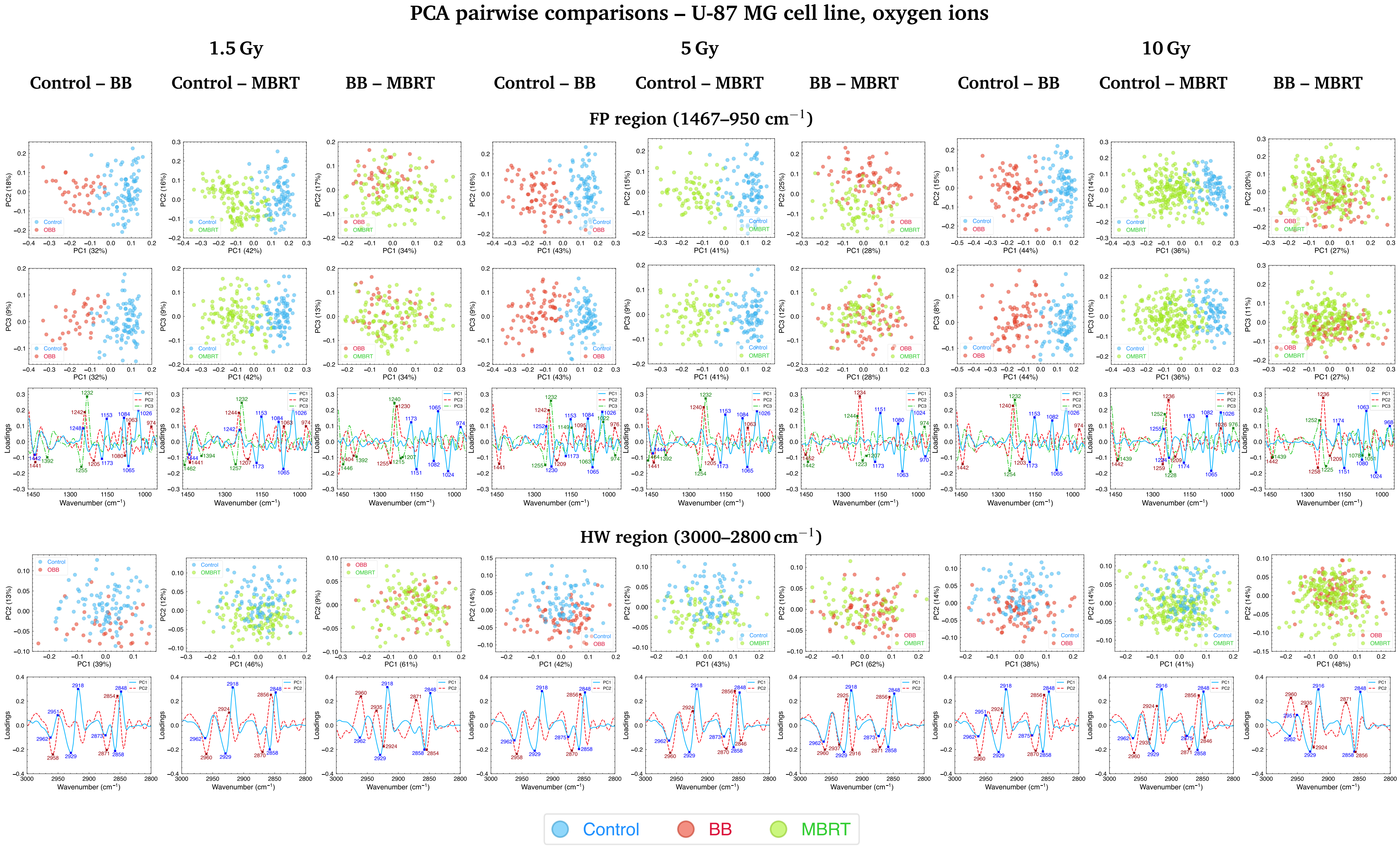

Figure S7. Pairwise PCA in the FP (1467–950 cm^{-1}, top) and HW (3000–2800 cm^{-1}, bottom) spectral regions of U-87 MG cells subjected to 1.5 Gy (left), 5 Gy (centre) and 10 Gy (right) oxygen irradiations. Each point of the PCA scores represents a cell spectrum, and colours correspond to the irradiation configurations: blue for Control, red for BB and green for MBRT. For each dose, Control–BB (first column), Control–MBRT (second column) and BB–MBRT (third column) pairwise analyses are included. Variances explained by the PCs are included in parentheses. In the loadings, the contribution of each spectral band to data separation along PC1, PC2 or PC3 is indicated by solid blue, red lines or green lines (respectively). The most relevant IR peaks contributing to the cluster delineation along PC1, PC2 or PC3 are indicated with blue, red or green labels and crosses (respectively). Indicated doses refer to the mean dose for both BB and MBRT configurations.

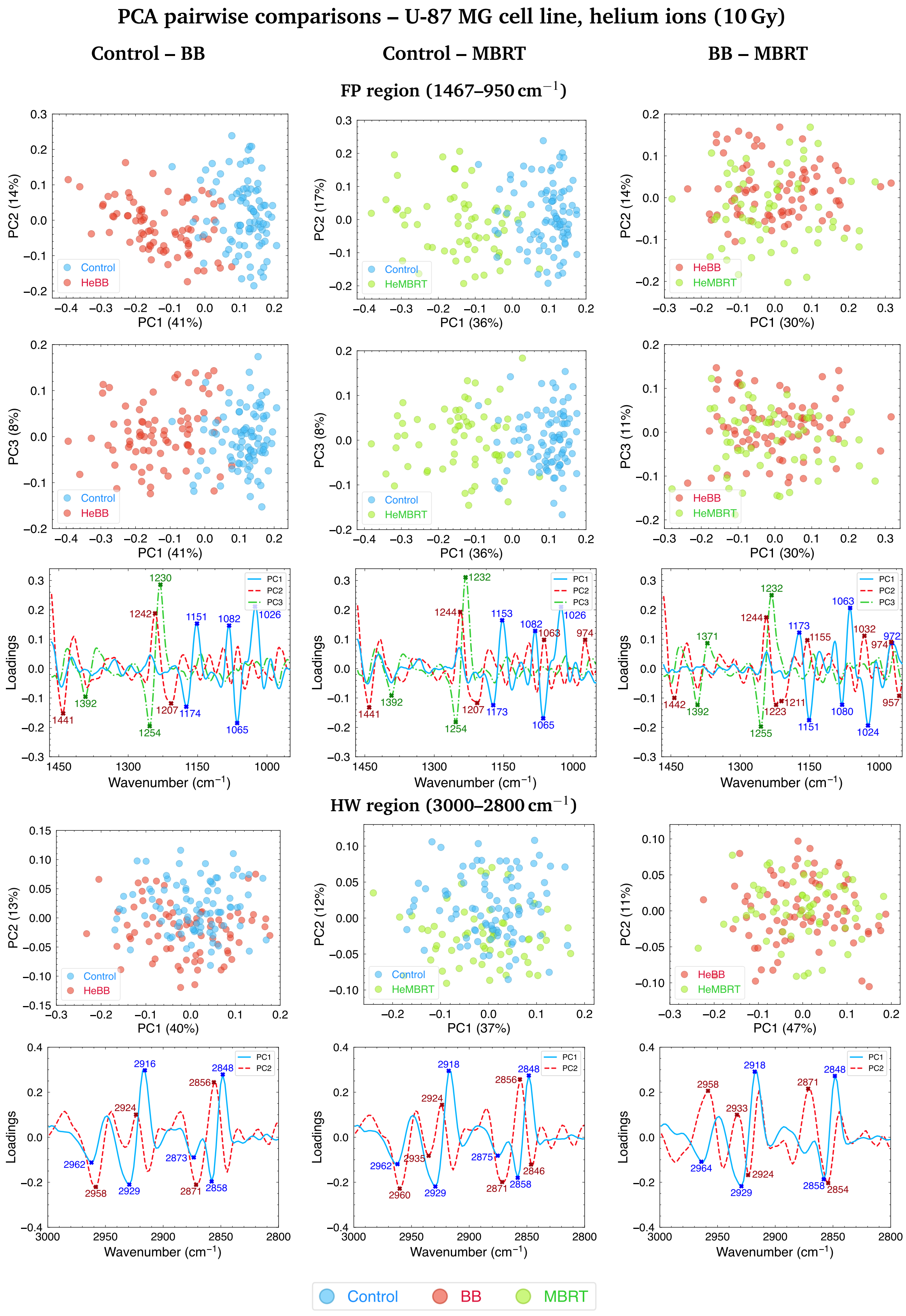

Figure S8. Pairwise PCA in the FP (1467–950 cm^{-1}, top) and HW (3000–2800 cm^{-1}, bottom) spectral regions of U-87 MG cells subjected to 10 Gy helium irradiations. Each point of the PCA scores represents a cell spectrum, and colours correspond to the irradiation configurations: blue for Control, red for BB and green for MBRT. For each spectral region, Control–BB (first column), Control–MBRT (second column) and BB–MBRT (third column) pairwise analyses are included. Variances explained by the PCs are included in parentheses. In the loadings, the contribution of each spectral band to data separation along PC1, PC2 or PC3 is indicated by solid blue, red lines or green lines (respectively). The most relevant IR peaks contributing to the cluster delineation along PC1, PC2 or PC3 are indicated with blue, red or green labels and crosses (respectively). A single mean dose of 10 Gy was studied for both BB and MBRT modalities.
