# Supplementary figures and images for: Revealing the biomolecular response of glioma cells to helium, carbon and oxygen minibeam radiation therapy using synchrotron-based infrared microspectroscopy
Source: Analyst. 2026 Jun 22;151(15):4424–42. doi: 10.1039/d5an01327e (PMC13285976; doi:10.1039/d5an01327e)

Carbon ions

Oxygen ions

1.5 Gy

5 Gy

10 Gy

1.5 Gy

5 Gy

10 Gy

FP region (1467–950  $\text{cm}^{-1}$ )

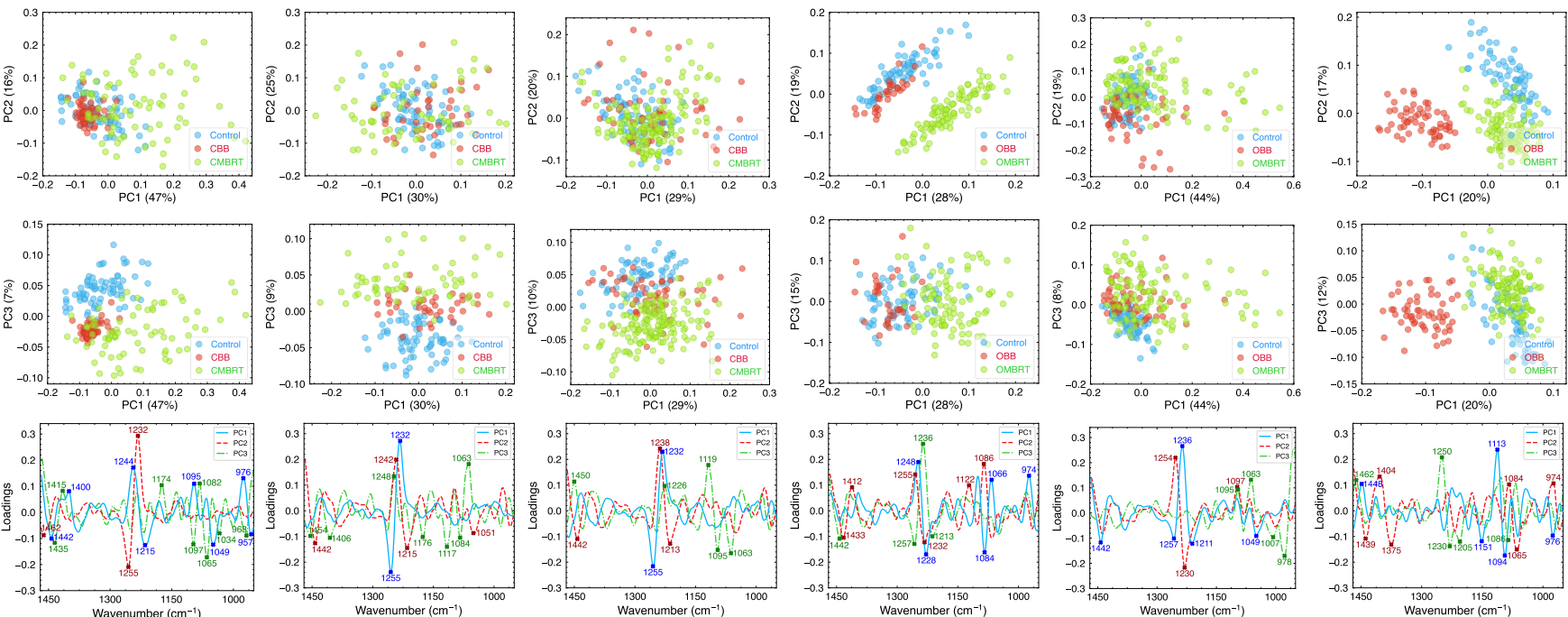

HW region (3000–2800  $\text{cm}^{-1}$ )

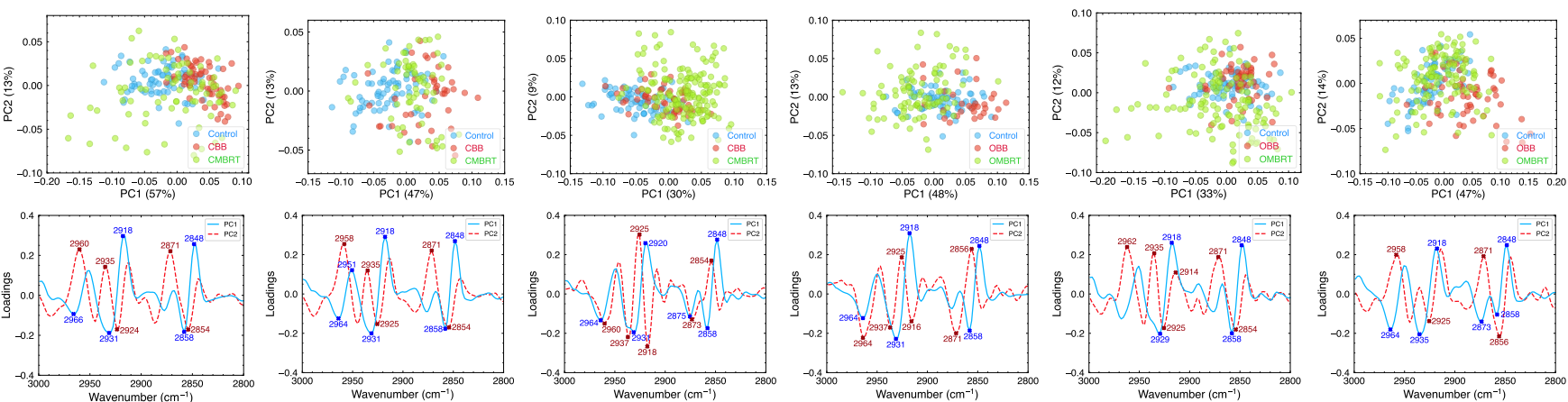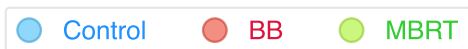

Supplement: AN-151-D5AN01327E-s004 [file AN-151-D5AN01327E-s004.pdf]

## Carbon ions

1.5 Gy

5 Gy

10 Gy

## Oxygen ions

1.5 Gy

5 Gy

10 Gy

## Helium ions

10 Gy

FP region (1467–950  $\text{cm}^{-1}$ )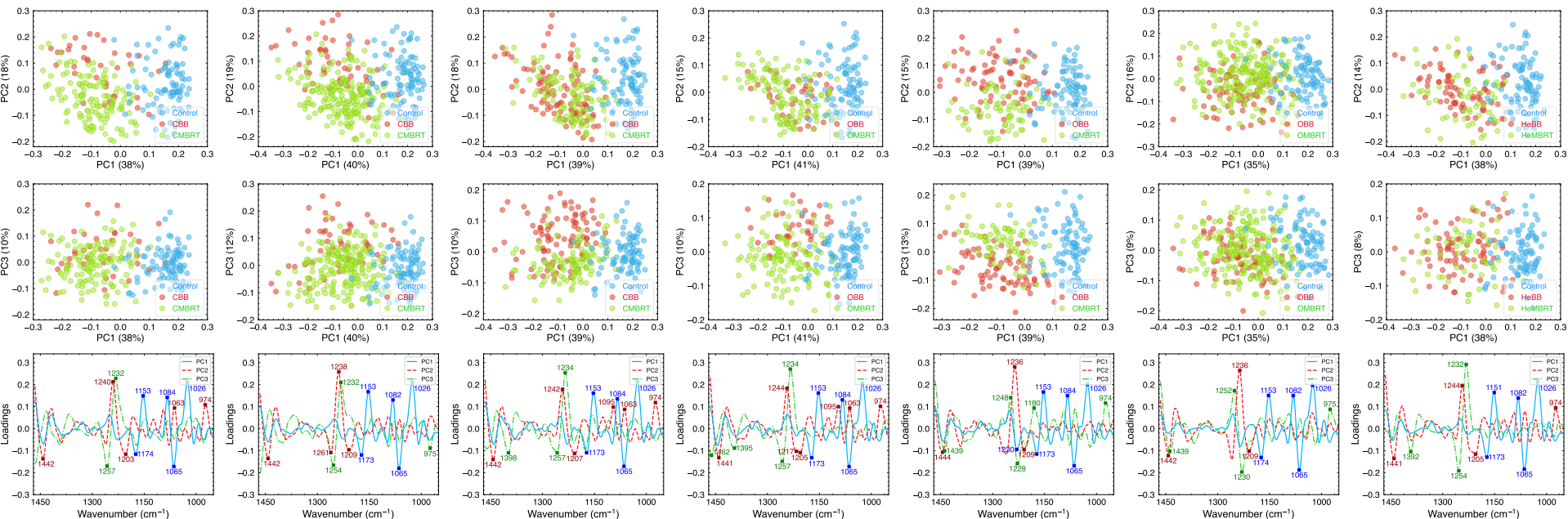HW region (3000–2800  $\text{cm}^{-1}$ )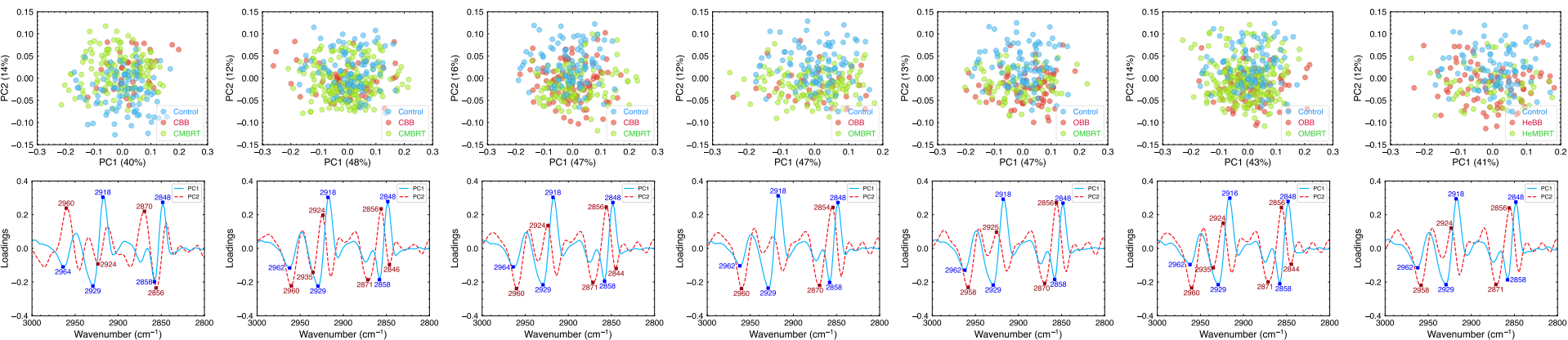

Supplement: AN-151-D5AN01327E-s008 [file AN-151-D5AN01327E-s008.pdf]

**Control – BB**

**Control – MBRT**

**BB – MBRT**

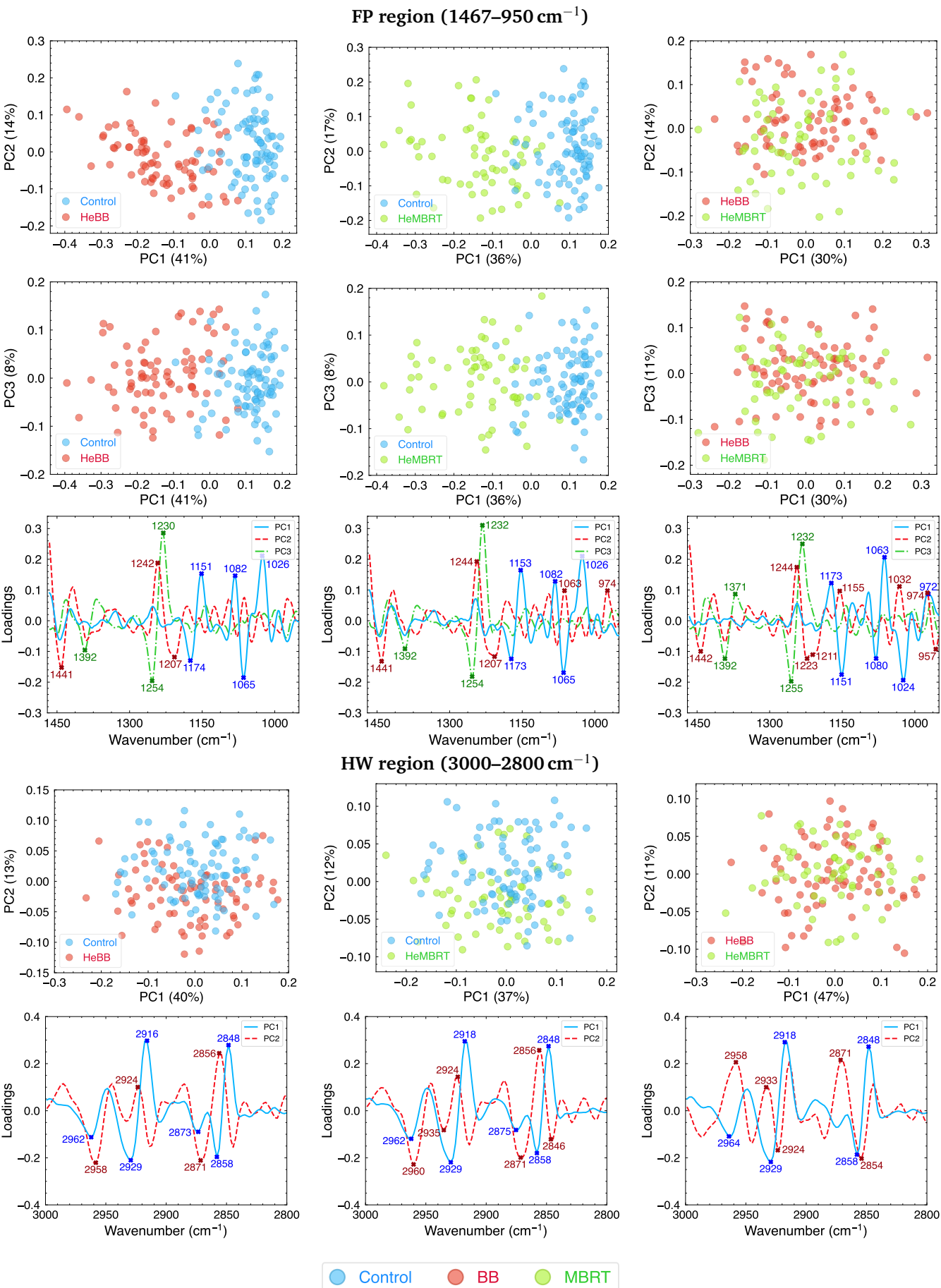

Supplement: AN-151-D5AN01327E-s011 [file AN-151-D5AN01327E-s011.pdf]
