# Supplementary material for: Revealing the biomolecular response of glioma cells to helium, carbon and oxygen minibeam radiation therapy using synchrotron-based infrared microspectroscopy
Source: Analyst. 2026 Jun 22;151(15):4424–42. doi: 10.1039/d5an01327e (PMC13285976; doi:10.1039/d5an01327e)

Supplementary Information (SI) for Analyst.
This journal is © The Royal Society of Chemistry 2026

PCA pairwise comparisons – F98 cell line, oxygen ions

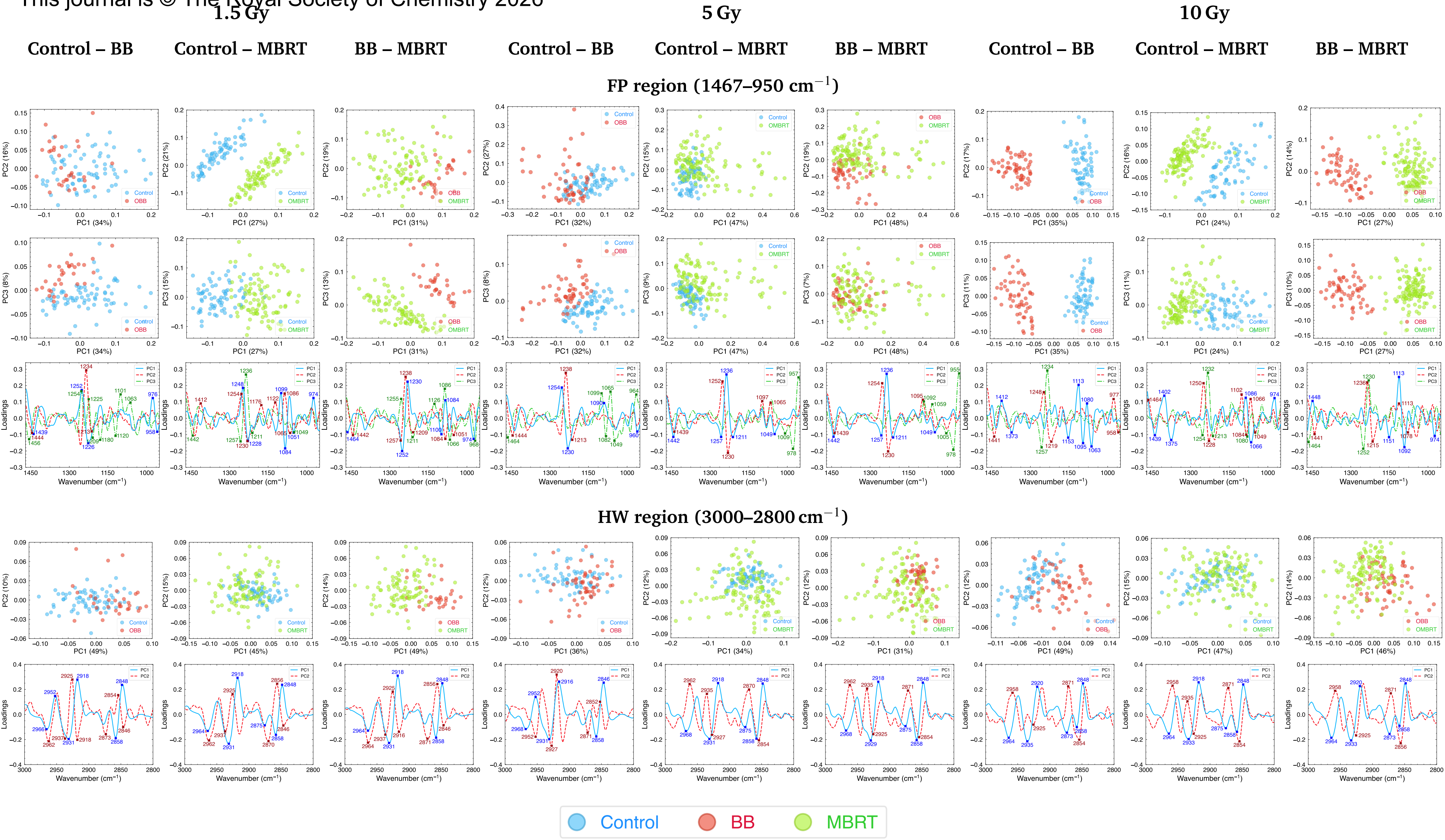

Supplement: AN-151-D5AN01327E-s006 [file AN-151-D5AN01327E-s006.pdf]
